# In vitro–in silico-based prediction of inter-individual and inter-ethnic variations in the dose-dependent cardiotoxicity of R- and S-methadone in humans

**DOI:** 10.1007/s00204-022-03309-y

**Published:** 2022-05-23

**Authors:** Miaoying Shi, Yumeng Dong, Hans Bouwmeester, Ivonne M. C. M. Rietjens, Marije Strikwold

**Affiliations:** 1 Division of Toxicology, Wageningen University, Stippeneng 4, 6708 WE Wageningen, The Netherlands; 2 NHC Key Laboratory of Food Safety Risk Assessment, Chinese Academy of Medical Sciences Research Unit (No. 2019RU014), China National Center for Food Safety Risk Assessment, Beijing, 100021 China; 3 Van Hall Larenstein University of Applied Sciences, 8901 BV Leeuwarden, The Netherlands

**Keywords:** Methadone, Human-induced pluripotent stem cell-derived cardiomyocytes (hiPSC-CM), Quantitative in vitro to in vivo extrapolation (QIVIVE), Inter-individual differences, Monte Carlo simulation, Physiologically based kinetic (PBK) modelling, New approach methodologies (NAM)

## Abstract

New approach methodologies predicting human cardiotoxicity are of interest to support or even replace in vivo-based drug safety testing. The present study presents an in vitro–in silico approach to predict the effect of inter-individual and inter-ethnic kinetic variations in the cardiotoxicity of R- and S-methadone in the Caucasian and the Chinese population. In vitro cardiotoxicity data, and metabolic data obtained from two approaches, using either individual human liver microsomes or recombinant cytochrome P450 enzymes (rCYPs), were integrated with physiologically based kinetic (PBK) models and Monte Carlo simulations to predict inter-individual and inter-ethnic variations in methadone-induced cardiotoxicity. Chemical specific adjustment factors were defined and used to derive dose–response curves for the sensitive individuals. Our simulations indicated that Chinese are more sensitive towards methadone-induced cardiotoxicity with Margin of Safety values being generally two-fold lower than those for Caucasians for both methadone enantiomers. Individual PBK models using microsomes and PBK models using rCYPs combined with Monte Carlo simulations predicted similar inter-individual and inter-ethnic variations in methadone-induced cardiotoxicity. The present study illustrates how inter-individual and inter-ethnic variations in cardiotoxicity can be predicted by combining in vitro toxicity and metabolic data, PBK modelling and Monte Carlo simulations. The novel methodology can be used to enhance cardiac safety evaluations and risk assessment of chemicals.

**Supplementary Information:**

The online version contains supplementary material available at 10.1007/s00204-022-03309-y.

## Introduction

Cardiotoxicity is an important endpoint in drug safety evaluation as it has been a leading cause of drug attrition during the development stage and leads to the withdrawal of marketed drugs (Ferri et al. 2013). Recently, we demonstrated that quantitative in vitro to in vivo extrapolation (QIVIVE) using physiologically based kinetic (PBK) modelling-based reverse dosimetry is an adequate approach to predict in vivo cardiotoxicity of racemic methadone (rac-methadone) in humans (Shi et al. 2020a). In this previous work, the cardiotoxic effects of rac-methadone on human-induced pluripotent stem cell-derived cardiomyocytes (hiPSC-CMs) were quantified in vitro using the multi-electrode array system. The obtained in vitro concentration–response curve for the field potential duration corrected for beat rate (FPDc), resembling the parameter observed in the human electrocardiogram (Zwartsen et al. 2019), was extrapolated to a predicted in vivo dose–response curve, which matched well with in vivo clinical data on rac-methadone-induced QTc prolongation.

Methadone is a prescription drug for the treatment of opioid addiction and chronic pain, which, however, has been associated with QTc interval prolongation in the clinic (Alinejad et al. 2015). Methadone is usually administered as the racemic preparation, a 1:1 mixture of the R- and S-enantiomer, with mainly the S-enantiomer being responsible for the cardiotoxic effects observed in vivo (Ansermot et al. 2010) and in vitro (Eap et al. 2007). Eap et al. (2007), reported that S-methadone showed a 3.5-fold higher potency than R-methadone in blocking the human ether-à-go-go-related gene (hERG) currents which play an important role in cardiac repolarization (Martin et al. 2004). Methadone is predominately cleared by hepatic cytochrome P450 (CYP) enzymes via *N*-demethylation and cyclisation to its primary metabolite 2-ethylidene-1,5-dimethyl-3,3-diphenylpyrrolidine (EDDP), which was found to be non-cardiotoxic in vitro at therapeutic relevant internal concentrations (Eap et al. 2007; Shi et al. 2020a). The major enzymes mediating the formation of EDDP have been identified in both in vitro and in vivo studies to be CYP2B6, CYP3A4 and to a lesser extent CYP2C19, with CYP2C19 and CYP2B6 showing stereoselectivity towards the conversion of R- and S-methadone, while CYP3A4 appeared to convert R- and S-methadone without stereoselectivity (Chang et al. 2011; Eap et al. 2007; Foster et al. 1999; Gerber et al. 2004; Kharasch 2017; Totah et al. 2007).

An increasing number of drug failures has been associated to unexpected extreme effects or inefficacy effects in clinical studies, pointing out the importance of studying inter-individual variation in responses to drug candidates and identifying covariates resulting in such variations (Tracy et al. 2016). Ethnic differences in demography, physiology and genetic background may affect the kinetic processes thereby contributing to uncertainty in the safety evaluation of compounds (Malinowski et al. 2008; Ning et al. 2017). Moreover, polymorphism in CYP enzymes is considered to be one of the most important factors contributing to the inter-individual variability in sensitivity towards compound exposure and thus needs to be incorporated in deciding on individual dosing regimens (Chiba et al. 2017; Zanger and Schwab 2013). The PBK model established in our previous work (Shi et al. 2020a) was defined for the Caucasian population as a whole, while inter-individual and inter-ethnic differences in kinetics were not yet considered. Large inter-individual variations in methadone pharmacokinetics have been reported to be the result of variability in the expression of the CYP isoforms involved in methadone metabolism (Eap et al. 2002). Given the highly polymorphic gene of CYP2B6 (Kharasch 2017; Zanger and Klein 2013) and the large variations between Caucasians and Chinese in the abundance of CYP3A4 (Barter et al. 2013), it is of interest to include such variabilities in the PBK model-based reverse dosimetry approach and predict their effects on the in vivo cardiotoxicity of R- and S-methadone.

PBK modelling and Monte Carlo simulations have been used to assess inter-individual variation in drug safety evaluations (Ito et al. 2017; Mehrotra et al. 2012). However, most studies involve in vivo data and specific dosing regimens, while the inter-individual variation on the whole population level for different dosing regimens was not quantitatively evaluated. In the safety assessment of chemicals, the International Programme on Chemical Safety (IPCS) has proposed the chemical-specific adjustment factor (CSAF) as a parameter to quantify inter-species or human inter-individual differences in toxicokinetics or toxicodynamics, and such a factor may equally well be defined for chemical drugs like methadone (IPCS 2005). The default uncertainty factor of 10 is set for human inter-individual differences with a subdivision for a factor of 3.16 accounting for human variability in toxicokinetics and 3.16 for variability in toxicodynamics (IPCS 2005).

Previously, a new approach methodology (NAM, EPA 2021) combining in vitro data, PBK modelling and Monte Carlo simulations has been used to predict inter-individual and/or inter-ethnic variations in in vivo toxicity for developmental toxicity of phenol (Strikwold et al. 2017) and liver toxicity of lasiocarpine (Ning et al. 2019). The aim of the present study was to demonstrate such an approach for the cardiotoxicity of R- and S-methadone, and to elucidate the consequences of inter-ethnic and inter-individual kinetic variations for the sensitivity towards these pharmaceuticals. To obtain this insight, PBK models for the two methadone enantiomers were developed and variations in their CYP-mediated metabolism were incorporated using two different approaches including (1) metabolic variation obtained from incubations with 25 Caucasian and 25 Chinese individual human liver microsomes (HLMs), and (2) reported variation in CYP abundances combined with Monte Carlo simulations. Ultimately, the maximum concentrations (C_max_) of R- and S-methadone in the heart venous blood of the Caucasian and Chinese populations were predicted, from which CSAFs were derived to describe the inter-individual kinetic variations within the different populations. Subsequently, the CSAFs were applied to the predicted in vivo dose–response curves obtained by reverse dosimetry of in vitro cardiotoxicity data to predict the toxicity for the most sensitive individuals within the populations based on which the safety in use of R- and S-methadone was discussed.

## Materials and methods

### Chemical and biological materials

Rac-methadone hydrochloride (≥ 98%, R-methadone: S-methadone 1:1), rac-EDDP perchlorate (≥ 98%, R-EDDP: S-EDDP 1:1), Tris (hydroxymethyl) aminomethane (Trizma^®^ base), and ammonium acetate were purchased from Sigma-Aldrich (Zwijndrecht, The Netherlands). The use of rac-methadone was in compliance with the registration (opium exemption license number 104783 03 WCO) at Farmatec (executive organization of the Ministry of Health, Welfare and Sport, The Hague, The Netherlands). Dimethyl sulfoxide (DMSO, 99.7%) was obtained from Merck (Schiphol-Rijk, The Netherlands). Acetonitrile (ACN, UPLC/MS grade) was obtained from Biosolve BV (Valkenswaard, The Netherlands). Reduced nicotinamide adenine dinucleotide phosphate (NADPH) regenerating system solution A and solution B were purchased from Corning (Woburn, MA, USA). Twenty-five individual Caucasian male human liver microsomes were obtained from XenoTech (Lenexa, USA). Twenty-five individual Chinese male human liver microsomes were purchased from PrimeTox (Wuhan, China). Detailed information of the human liver microsome donors is shown in Table S1 in the supplementary materials 1.

### General outline of PBK modelling and Monte Carlo simulation

To investigate the inter-individual and inter-ethnic variations in the cardiotoxicity of R- and S-methadone, the present study included the following steps: (1) Generation of information on the metabolic variation in CYP-mediated conversion of R- and S-methadone using two approaches. In the first approach information on the metabolic variation of R- and S-methadone conversion was generated from in vitro kinetic experiments using 25 Chinese and 25 Caucasian individual liver microsomes, while in the second approach information on variation in the metabolism was obtained based on reported kinetic constants for R- and S-methadone of recombinant CYP isoforms (rCYPs) together with reported variation in their expression in the Caucasian and the Chinese population. (2) Development and evaluation of PBK models for R- and S-methadone using the metabolic parameters obtained from the two approaches. (3) Integrating metabolic variations, PBK modelling and the Monte Carlo simulation to predict inter-individual and inter-ethnic variations in the kinetics of R- and S-methadone and the calculation of CSAFs for human kinetics. (4) PBK modelling-based reverse dosimetry and dose–response analysis of R- and S-methadone-induced cardiotoxicity for the average and the sensitive populations in the Caucasian and Chinese populations for the safety evaluation of R- and S-methadone.

### Generation of metabolic variation data in the conversion of R- and S-methadone toward R- and S-EDDP

#### In vitro incubations with 25 Caucasian and 25 Chinese individual liver microsomes

In vitro incubations with 25 male Caucasian and 25 male Chinese individual liver microsomes were performed as previously described by Shi et al. (2020a). Based on the IPCS guideline, this number of individual microsomes is sufficient to accurately measure the central tendency of the whole population (IPCS 2005). In brief, incubation samples with a final volume of 160 µl were prepared in 0.1 M Tris–HCl (pH 7.4) containing the NADPH regeneration system (final concentrations 1.3 mM NADP^+^, 3.3 mM glucose-6-phosphate, 0.4 U/ml glucose-6-phosphate dehydrogenase and 3.3 mM magnesium chloride) and rac-methadone at seven final concentrations ranging from 25 to 1500 µM added from a concentrated stock solution of 100 mM in water. Control samples were prepared in the same way, but in the absence of NAPDH-regeneration system which was replaced with Tris–HCl. Samples were pre-incubated at 37 °C for one minute and the reactions were started by adding individual human liver microsomes at a final concentration of 0.5 mg/ml microsomal protein. After 40 min incubation at 37 °C, 40 µl ice-cold ACN were added to terminate the reaction. Then samples were put on ice for 20 min and centrifuged at 18,000×*g* for 5 min at 4 °C to precipitate microsomal proteins. The supernatant was collected and diluted 2 to 10 times with ACN for the quantification of R- and S-EDDP by liquid chromatography–mass spectrometry (LC–MS/MS) as described in the “LC–MS/MS analysis” section. Under these conditions, the reaction rate was shown to be linear with respect to incubation time and microsomal protein concentration. Given that no gender differences in metabolism of methadone have been reported in the literature (Graziani and Nisticò 2015) and that the average of catalytic efficiency for rac-methadone metabolism obtained from 25 male Caucasian HLMs was comparable with the one obtained from the mix-gender microsomal pool of 150 donors (Shi et al. 2020a), the metabolic variations derived from male individuals are expected to be comparable to those for mixed gender.

The metabolic parameters including the apparent maximum reaction rate (*V*_max_) and the apparent Michaelis–Menten constant (*K*_m_) for the methadone dependent formation of R- and S-EDDP were defined using GraphPad Prism 5.0 (GraphPad Software Inc., San Diego, USA.) to fit the data obtained from the in vitro microsomal incubations to the Michaelis–Menten Eq. (1):

1 $$V = \frac{{V_{\max } * [S]}}{{K_{m} + [S]}},$$

where [S] is the substrate (R- or S-methadone) concentration (µM) and v is the rate of R- or S-EDDP formation (nmol/min/mg microsomal protein). The in vitro catalytic efficiency expressed in µl/min/mg microsomal protein was calculated by dividing *V*_max_ by *K*_m_. Data were collected from two independent experiments and each data point is presented as the mean value ± SD. The mean values and the coefficient of variations (CVs) of *V*_max_ and *K*_m_ were calculated using Microsoft Excel 2016 (Microsoft Corporation, Washington, USA).

#### Kinetic constants for R- and S-methadone conversion by rCYPs and variations in CYP abundances in the Caucasian and the Chinese population

CYP3A4, CYP2B6 and CYP2C19 are the major CYPs involved in the metabolism of both methadone enantiomers (Chang et al. 2011; Totah et al. 2007), and their kinetic constants (*V*_max, CYP_ and *K*_m, CYP_) for the conversion of R- and S-methadone toward R- and S-EDDP were obtained from the study of Totah et al. (2007). These kinetic constants were determined using Baculovirus-insect cells (Supersomes) expressing recombinant CYP2B6, CYP2C19 and CYP3A4 and the kinetic constants for methadone conversion by each CYP are shown in Table 1. To correct for the differences between the activity of the CYPs in the rCYP system and the HLM system, the reported *V*_max_ for Supersomes (*V*_max, CYP_, pmol/min/pmol CYP) were scaled to the *V*_max_ value for microsomes (*V*_max, CYP in HLM_, pmol/min/mg protein) using the following Eq. (2):

2 $$V_{{\max ,{\text{CYP}}\,\,{\text{in}}\,\,{\text{HLM}}}} \, = \,V_{{\max ,{\text{CYP}}}} *{\text{ISEF}}*{\text{CYP}}\,{\text{abundance}},$$

where ISEF is the CYP isoform specific inter-system extrapolation factor to correct for differences in the intrinsic activity between Supersomes and microsomes taking into account the relative abundance of the respective CYP in HLM (Proctor et al. 2004). CYP abundance (pmol/mg protein) is the expression level of the individual CYP present in HLM samples which were collected from the literature (Table 1). The ISEFs for the three CYPs were calculated using the following Eq. (3) for each methadone enantiomer:

3 $${\text{ISEF = }}\frac{{{\text{CL}}_{{_{{{\text{int}}}} {\text{, CYP in HLM}}}} }}{{{\text{CL}}_{{\text{int, CYP }}} {\text{* CYP abundance}}}},$$

where CL_int, CYP in HLM_ (µl/min/mg protein) represents in vitro intrinsic clearance of R- or S-methadone by each CYP in HLM, which were determined by multiplying the in vitro total CYP-mediated intrinsic clearance for the respective methadone enantiomer in Caucasian or Chinese HLM (CL_int, HLM_) measured in this study by the relative contribution of the respective CYP to the total CYPs in the HLM (f_m, CYP_). This relative contribution, defined as fraction metabolised by each CYP of the total in vitro metabolic clearance amounted to 0.44, 0.09 and 0.46 (R-methadone), and 0.59, 0.09 and 0.32 (S-methadone) for CYP2B6, CYP2C19 and CYP3A4, respectively (Totah et al. 2008). These *f*_m, CYP_ values were obtained by the incubation of Caucasian HLM with inhibitors of the specific CYP isoforms (Totah et al. 2008). CL_int, CYP_ (µl/min/pmol CYP) represents the in vitro intrinsic clearance of the methadone enantiomers by each CYP reported by Totah et al. (2007). CL_int, CYP in HLM_ and CL_int, CYP_ were calculated from the enzyme kinetic parameters (*V*_max_/*K*_m_) determined in HLM and the Supersomes, respectively. The calculated ISEFs for the Caucasians and Chinese are shown in Table 1. Due to lacking information about the *f*_m, CYP_ for the Chinese population, the *f*_m, CYP_ of the Caucasian was used to calculate ISEF values for the Chinese population. The detailed information used for calculation of the ISEF and *f*_m, CYP_ can be found in Table S2 and S3 in the supplementary materials 1.

**Table 1:** Summary of kinetic parameters for conversion of R- and S- methadone to R- and S-EDDP by CYP2B6, CYP2C19 and CYP3A4 and the hepatic CYP abundances, genotypes and corresponding phenotype frequencies

CYP	R-methadone				S-methadone				Caucasian			Chinese		
	V_max, CYP_ (pmol/min/pmol CYP)^a^	K_m, CYP_ (µM)^a, b^	ISEF (Caucasian/Chinese)	Catalytic efficiency (µl/min/mg protein) (Caucasian/Chinese)	V_max, CYP_ (pmol/min/pmol CYP)^a^	K_m, CYP_ (µM)^a, b^	ISEF (Caucasian/Chinese)	Catalytic efficiency (µl/min/mg protein) (Caucasian/Chinese)	Phenotype (frequency)	Mean abundance (μ_x_, pmol/mg protein)	CV (%)	Phenotype (frequency)	Mean abundance (μ_x_, pmol/mg protein)	CV (%)
2B6	36	60	0.13/0.072	1.16/0.22	15	16	0.13/0.049	1.81/0.24	EM (0.89)^c^	17^c^	122^c^	EM (0.95)^d^	5.3^c^	198^c^
									PM (0.11)^c^	6^c^	200^c^	PM (0.05)^d^	1.9^c^	200^c^
2C19	22	97	0.1/0.047	0.25/0.05	8	125	0.39/0.13	0.27/0.04	General group	11^e^	82^e^	EM (0.87)^c^	4.4^c^	39^c^
3A4	43	137	0.04/0.0062	1.21/0.23	46	149	0.03/0.0034	0.97/0.13	General group	93^e^	81^e^	EM (1)^d^	120^f^	33^f^

*ISEF* inter-system extrapolation factors, *EM* extensive metabolizer, *PM* poor metabolizer

^a^Values were obtained from the global analysis of racemate metabolism reported in Totah et al. (2007)

^b^The reported dissociation constant (K_s_) was used as K_m_ assuming rapid equilibrium for formation of the enzyme methadone complex (dissociation of enzyme methadone complex rate constant, k_2_ >  > the complex to the products conversion rate constant, k_3_)

^c^Obtained from the Simcyp simulator V18 Release 1 (Certara)

^d^Based on Guan et al. (2006)

^e^Values were summarized by Achour et al. (2014) from different studies

^f^Reported by Shu et al. (2000)

### LC–MS/MS analysis

The chiral separation of R- and S-methadone and their metabolites and quantification was performed by LC–MS/MS analysis using a Shimadzu Nexera XR LC-20AD SR UPLC system coupled with a Shimadzu LCMS-8045 mass spectrometer (Kyoto, Japan). Samples were loaded on a CHIRALPAK^®^ AGP column (100 × 4 mm 5 μm Analytical Column M) and CHIRALPAK^®^ AGP pre-column (0.4 cm × 1 cm 5 μm) with an injection volume of 1 µl. A Shimadzu LCMS-8045 triple quadrupole with electrospray ionization interface was used to perform the MS–MS analysis. The instrument was operated in positive mode in the multiple reaction monitoring (N2 collision gas) mode. The multiple reaction monitoring of m/z 310.20 (MH^+^) to 265.15 (CE: – 15 V), m/z 310.20 (MH^+^) to 105.05 (CE: – 28 V) and m/z 310.20 (MH^+^) to 77.15 (CE: – 51 V) were used to analyse R- and S-methadone. The m/z 278.10 (MH^+^) to 234.20 (CE: – 31 V), m/z 278.10 (MH^+^) to 249.15 (CE: – 25 V) and m/z 278.10 (MH^+^) to 186.15 (CE: – 38 V) were used to analyse R- and S-EDDP. The MRMs were selected based on previous studies (Malinowski et al. 2008; Chang et al. 2011). For optimal chiral separation, an isocratic mobile phase of 10 mM ammonium acetate (pH = 7.0): ACN (85: 15, v/v) with a flow rate of 1 ml/min was applied. The temperature of the column was kept at 20 °C. The retention times for R- EDDP, S-EDDP, R-methadone and S-methadone were 10.1, 12.9, 14.5 and 19.6 min, respectively, determined using commercially available reference compounds. Quantification was based on comparison of the respective peak areas of the total ion chromatogram (TIC) to the TIC peak areas of corresponding linear calibration curves obtained from standards prepared in ACN using the reference compounds (*R*^2^ > 0.999), using Postrun analysis in the software LabSolution (Shimadzu).

### Development of the PBK models of R- and S-methadone for the Caucasian and Chinese population

**Fig. 1:**
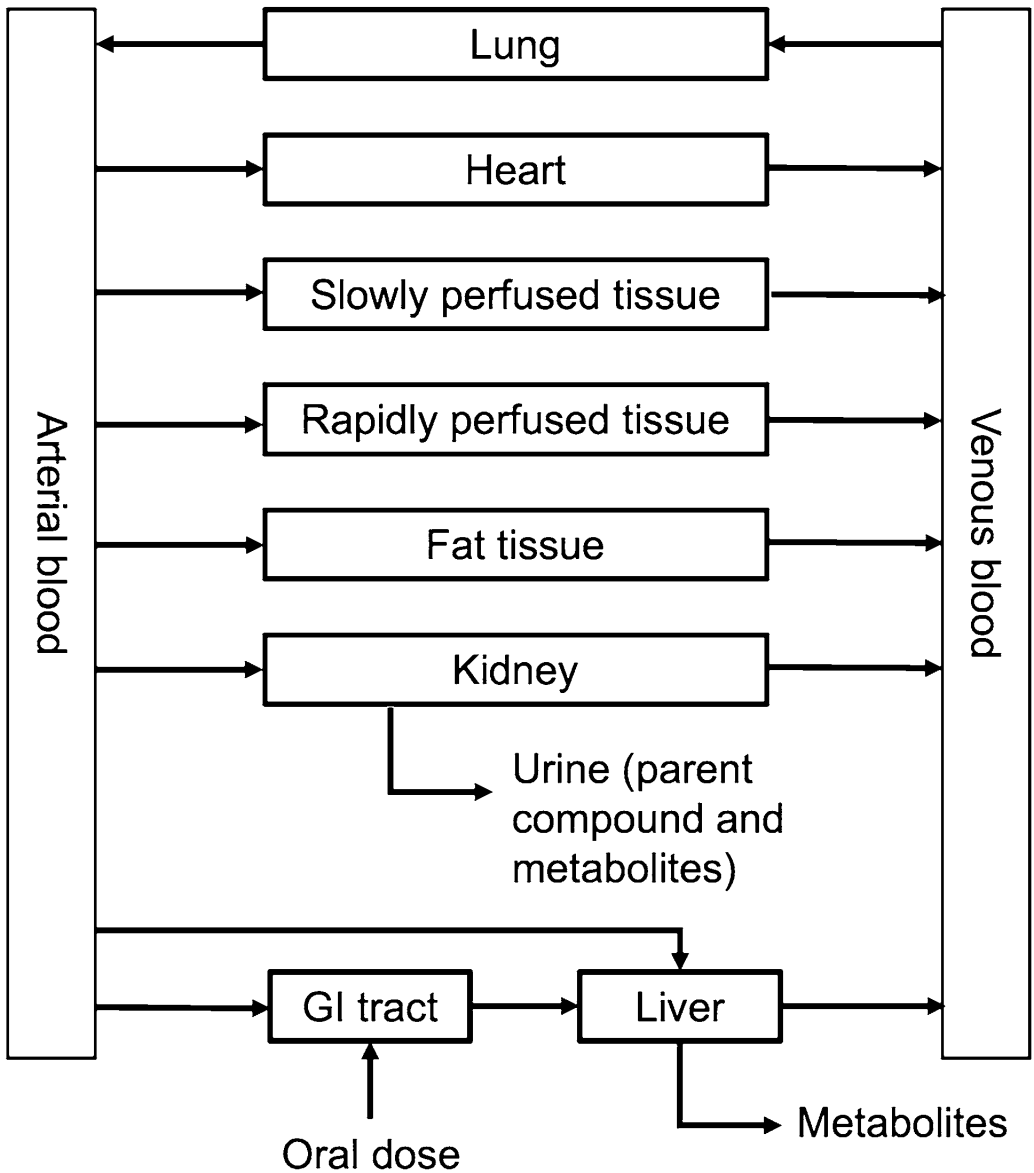
Schematic diagram of the PBK model of R- and S-methadone

The PBK model of rac-methadone developed in the study of Shi et al. (2020a) was adjusted to describe the absorption, distribution, metabolism and excretion (ADME) of R- and S-methadone in the Caucasian and Chinese populations in the Berkeley Madonna software (version 8.3.18, UC Berkeley, CA, USA) applying Rosenbrock’s algorithms for solving stiff systems. Figure 1 presents the schematic diagram of the PBK model and the compartments relevant for the ADME characteristics. The PBK model is developed for repeated dosing given that methadone is usually administrated daily.

Human physiological parameters used in the PBK model for the Caucasian population were obtained from Brown et al. (1997) and for the Chinese population from NHFPC (2007a, b; 2014) (Table S4 in supplementary materials 1). The volume of the arterial, venous blood and the blood flow to the heart are not available for the Chinese population and were assumed to be the same as the ones for the Caucasian, which is regarded suitable since these parameters are not influential on the model outcome (see results in sensitivity analysis). The physicochemical parameters of R- and S-methadone are presented in Table S5 in supplementary materials 1. Given that no chiral difference was reported in absorption related parameters (Ke et al. 2014; Badhan et al. 2019), values for these parameters were similar for both enantiomers, including a mean oral absorption rate constant (ka) value of 0.59 and a mean fraction absorbed (Fa) value of 0.88 obtained from studies on rac-methadone (Foster et al. 2000; Ke et al. 2014).

Tissue: blood partition coefficients (P) of R- and S-methadone were obtained by dividing tissue: plasma partition coefficients by the corresponding blood/plasma ratio to correct for the differences in the distribution of the compounds in blood and plasma. The blood/plasma ratio value of 0.7 reported by Hsu et al. (2013) was used and assumed to be similar for the two enantiomers (Badhan et al. 2019). The tissue: plasma partition coefficients of the two enantiomers (Table S5 in the supplementary materials 1) were estimated using a QIVIVE tool (version 1.0) from Wageningen Food Safety Research (WFSR 2020). The fraction unbound in plasma (*f*_u,p_), lipophilicity (logP) and acid–base properties (pKa) were used as the input for the algorithms of Berezhkovskiy (2004). The two enantiomers have the same logP and pKa values, amounting to 3.93 and 9.2, respectively (Ke et al. 2014; Gerber et al. 2004). The mean f_u,p_ values were obtained from several studies, amounting to 0.16 for R-methadone and 0.12 for S-methadone as reported by Ke et al. (2014).

As described in our validated PBK model for rac-methadone (Shi et al. 2020a), liver was considered as the metabolizing organ. The average values of kinetic constants (*V*_max_ and *K*_m_) obtained from incubations with ethnic-specific individual microsomes were used to define the metabolism of R- and S-methadone in the two populations, applying Michaelis–Menten kinetics. Besides, the metabolism of R- and S-methadone at the microsomal level was defined using reported rCYPs kinetic data of CYP2B6, CYP2C19 and CYP3A4. The enantiomeric interactions observed in the racemate metabolism using in vitro incubation of rCYPs (Totah et al. 2007) were included in the current model where the algorithms for the rate of R- and S-EDDP formation were described by two-substrate, two-site models with the competitive inhibition enabling the homotropic and heterotropic binding. The algorithms were taken from equations reported in Totah et al. (2007) in which enantiomeric interactions were described for the CYP2B6, CYP2C19 and CYP3A4 separately (model equations are shown in supplementary materials 2). Predicted blood kinetics were not distinctive between enantiomeric interaction equations and the Michaelis–Menten equation when both equations were modelled in the rCYP-based PBK model (data are not shown, both model equations are shown in supplementary materials 2), suggesting that predictions with the HLM PBK model without interaction are valid as well. The in vitro *V*_max_ values were scaled to the in vivo situation by using a microsomal protein per gram of liver (MPPGL) value of 32 mg/g for the Caucasian population (Barter et al. 2007) and a value of 39.46 mg/g for the Chinese population (Zhang et al. 2015b).

Besides metabolism, urinary excretion significantly contributes to the elimination of methadone (Lugo et al. 2005), and thus the urinary excretion was included in the model. The renal clearance values (RCL) were set at 1.8 l/h for R-methadone and 1.1 for S-methadone as reported in the study of Ke et al. (2014).

### Sensitivity analysis and evaluation of the PBK model for R- and S-methadone

The sensitivity analysis and model evaluation were performed for the PBK model with the average of the *V*_max_ and *K*_m_ values of the 25 individual HLMs and the PBK model with rCYPs defined metabolism data.

The influence of model parameters on the predicted R- and S-methadone *C*_max_ in the heart venous blood during the steady-state phase was identified by performing a local parameter sensitivity analysis. The sensitivity coefficient (SC) was determined according to the following equation:

4 $${\text{SC}} = { }\frac{{\left( {C{^{\prime}} - C} \right)}}{{\left( {P{^{\prime}} - P} \right)}}*\frac{P}{C},$$

where *C* represents the initial value of the model output, *C*’ is the model output after a 1% increase in an individual model parameter value. Similarly, *P* stands for the initial parameter value and *P*’ is the parameter value after a 1% increase. Only parameters with a SC > 0.1 are considered further, indicating a large impact on the model output (Rietjens et al. 2011). The sensitivity analysis was carried out for both the Caucasian and Chinese PBK model with the respective mean body weight of 70 kg (Brown et al. 1997) and 58.5 kg (NHFPC 2007a), and for oral daily doses of 20 and 200 mg rac-methadone (10 and 100 mg of each enantiomers) for 30 days, as previously described (Shi et al. 2020a).

The performance of the developed PBK models for R- and S-methadone was evaluated by comparing the predicted blood concentrations and area under the curve (AUC) values of the enantiomers to the respective in vivo data on these parameters obtained in clinical studies (Foster et al. 2000; Garimella et al. 2015; Liu et al. 2007) where the time dependent concentrations of R- and S-methadone were measured in plasma. The reported plasma-based kinetics of R- and S-methadone were extracted using GetData Graph Digitizer 2.26 and converted to blood-based kinetics with multiplication by the respective blood/plasma ratio values. The specifications of in vivo studies are summarized in Table S6 and S7 in the supplementary materials 1. Body weight, the oral dose and exposure duration were chosen to match the conditions used in the clinical studies for the PBK model evaluation. The performance of the enantiomeric PBK model for the Chinese was based on this evaluation of the model for the Caucasians since no in vivo kinetic data on dose-dependent blood or plasma levels of methadone enantiomers are available for the Chinese population.

### Prediction of inter-individual and inter-ethnic variations applying individual PBK models and PBK modelling combined with Monte Carlo simulations

The sensitivity analysis revealed that metabolic parameters are highly influential on the model output. To predict the influence of metabolic variations on the inter-individual and inter-ethnic differences in formation of R- and S-EDDP, two approaches were applied. In the first approach, 25 Caucasian and 25 Chinese individual PBK models were built by integrating the metabolic parameters obtained from the incubation of individual microsomes, to enable the prediction of *C*_max_ in the heart venous blood of the two enantiomers at a clinically relevant daily dose of 30 mg of each enantiomer administered for 30 consecutive days for each individual applying a mean body weight of 70 kg for Caucasian and 58.5 kg for Chinese (Brown et al. 1997; NHFPC 2007a).

In the second approach, the Monte Carlo simulation was performed together with PBK modelling to simulate the variation in the *C*_max_ in the heart venous blood of R- and S-methadone in the Caucasian and the Chinese population. Monte Carlo simulations of the respective PBK models were run for each enantiomer in the two populations with a daily dose of 30 mg of each enantiomer administered for 30 days applying a mean body weight of 70 kg for Caucasian and 58.5 kg for Chinese (Brown et al. 1997; NHFPC 2007a). To simulate the metabolic variation in R- and S-methadone formation, parameters for which random values were taken from the parameters’ log-normal distribution for Monte Carlo simulation were the CYP abundances in the Caucasian and Chinese population. The distribution in the CYP abundances were defined by their mean value and CV in the Caucasian and Chinese population. For that purpose, lognormally distributed CYP abundances were transformed to a normally distributed variable *ω* with the mean (*µ*_*ω*_) and standard deviation (*σ*_*ω*_) using the following equations (Zhang et al. 2007):

5 $$\mu_{\omega } {\text{ = ln}}\left( {\mu_{x} /\sqrt {{\text{1 + CV}}_{x}^{{2}} } } \right),$$

and

6 $$\sigma_{\omega }^{{2}} {\text{ = ln(1 + CV}}_{x}^{{2}} {)},$$

where *µ*_*x*_ is the mean of CYPs abundances obtained from literature (Table 1). CV_*x*_ is the coefficient of variation of non-transformed CYP abundances. Monte Carlo simulations were performed in Berkeley Madonna (version 8.3.18, UC Berkeley) using the parameter plot function. Individuals with CYP abundances that were three times the standard derivation higher or lower than the mean values were excluded from the Monte Carlo simulation (Ning et al. 2019; Strikwold et al. 2017).

For the distribution of the CYP2B6 abundance in the Caucasian and the Chinese population two phenotypes were distinguished, namely the extensive metabolisers and the poor metabolizers using values reported in the Simcyp simulator V18 Release 1 (Certara, Sheffield, UK). For CYP2C19 and CYP3A4, the CYP abundance values for the general Caucasian population reported by Achour et al. (2014), without phenotype specification were used, while for the Chinese population, the abundance distribution for the extensive metabolisers was used given the absence of distribution data for the general Chinese population together with high frequency of CYP2C19 and CYP3A4 extensive metabolisers in Chinese (Barter et al. 2013). The hepatic CYP abundances (mean and CV), genotypes and corresponding phenotype frequencies obtained from several studies are summarized in Table 1. Given that different phenotypes of CYP2B6 were integrated in the simulation, two separate Monte Carlo analyses were performed for CYP 2B6 extensive metabolisers and poor metabolizers, respectively. 15,000 Simulations were run to predict the probability distribution of *C*_max_ in the heart venous blood of R- and S-methadone for each CYP2B6 phenotype population. The distribution parameters for the whole population were calculated by using the weighted average parameters obtained in each phenotype population. Weighting was based on the extensive metabolisers and poor metabolizers phenotype frequencies in the two population as shown in Table 1. In the model simulation, the parameters were allowed to vary independently from each other. The summary of distribution parameters is shown in Table S8 in the supplementary materials 1. Model codes of Monte Carlo simulation are provided in the supplementary materials 3. In addition, Monte Carlo simulations were performed taking into account the variability of additional model parameters that appeared to influence the kinetics of the predicted blood concentrations of the methadone enantiomers (see sensitivity analysis in the Results section). These factors were (i) body weight, (ii) the oral fraction absorbed, and (iii) the fraction unbound in plasma that was shown before to have a strong influence on the predicted dose–response curves in humans (Shi et al. 2020a). The coefficients of variation for these three parameters were assumed to be 0.3 representing a moderate level of variation (Covington et al. 2007). Statistical analysis of the population distributions obtained with Monte Carlo simulations was performed in GraphPad Prism 5.0 (GraphPad Software Inc., San Diego, USA) calculating the geometric mean (GM), geometric CV, the 95th and 99th percentile of the *C*_max_ in the heart venous blood of R- and S-methadone for the two populations.

### Derivation of CSAF

Given that the IPCS (2005) guidance document recommends several options for the calculation of CSAFs, different approaches were applied for deriving CSAFs to provide comprehensive information for the risk assessment of methadone. First, the CSAF values for both the Caucasian and the Chinese population were derived by dividing the 95th and the 99th percentile of the unbound *C*_max_ in the heart venous blood of R-and S-methadone by the GM of the unbound *C*_max_ of R-and S-methadone. Additionally, the Chinese population was considered as the sensitive group, and the CSAF values were calculated by dividing the 95th and 99th percentile of the unbound *C*_max_ in the heart venous blood of both enantiomers in the Chinese population by the GM of the unbound *C*_max_ of the respective enantiomers in the Caucasian population (IPCS 2005). The calculations were performed in two ways considering variations in (1) metabolism only, and (2) multiple factors including metabolism, body weight, the oral fraction absorbed and fraction unbound in plasma. The CSAF values (based on variations in multiple factors) calculated based on the 99th percentile in each population were used to generate in vivo dose–response curves of R- and S-methadone for sensitive groups (99th percentile of *C*_max_) in each population as outlined in the next section. In the present study, definition of the sensitive groups is based on differences between individuals in methadone kinetics and does not cover any dynamic variation.

### PBK modelling-based reverse dosimetry and Benchmark dose analysis of in vivo cardiotoxicity predictions

In our previous study (Shi et al. 2020a), PBK model-based reverse dosimetry was applied to translate in vitro concentration–response curves for rac-methadone-induced cardiotoxicity to in vivo dose–response curves for QTc prolongation. A similar approach was applied to predict the in vivo cardiotoxicity of methadone enantiomers for the average and sensitive groups in Caucasian and Chinese populations. To this end, the in vitro cardiotoxic effects of rac-methadone on the FPDc measured in hiPSC-CMs using the multi-electrode array technique (Shi et al. 2020a) were used to derive concentration–response curves for R- and S-methadone. Given that S-methadone blocked hERG currents 3.5-fold more potently than R-methadone (Eap et al. 2007), the responses induced by rac-methadone were with the ratio of 1: 3.5 proportionally distributed to responses of R- and S-methadone, respectively. Subsequently, the *f*_*u*_ value of 0.79 for rac-methadone in the in vitro medium of hiPSC-CM multi-electrode array assay (Shi et al. 2020a), assumed to be the same for R- and S-methadone, was used to calculate unbound in vitro R- and S-methadone concentrations which were set equal to the unbound steady-state *C*_max_ of R- and S-methadone in the heart venous blood of the PBK model using the average of individual HLMs as specified by Shi et al. (2020a). Reverse dosimetry on each concentration-effect level tested in the hiPSC-CMs was performed using the PBK models for the average Caucasian and the average Chinese population using the average *V*_max_ and *K*_m_ values obtained from incubations with the 25 Caucasian and 25 Chinese human liver microsomes, generating in vivo dose–response data for R- and S-methadone. From this, the dose–response curves for R- and S-methadone were defined for the average Caucasian and the average Chinese population. The dose–response curves for sensitive groups in the Caucasian and the Chinese population were obtained by applying the respective CSAFs (calculated using the 99th percentile of *C*_max_, based on variations in multiple parameters) to the dose–response curves of the average populations given that no saturation occurs at the higher doses.

Benchmark dose (BMD) analysis of the predicted dose–response curves for R- and S-methadone was performed to obtain a benchmark dose resulting in 10% cardiotoxic effect (BMD_10_) for the general and sensitive groups in the Caucasian and Chinese population, where the FPDc derived in vitro, can be regarded a representative endpoint for the QTc interval in the human electrocardiogram. An effect size of 10% was chosen considering the physiological and statistical meaning of the abnormal QTc prolongation as previously described (Shi et al. 2020a). The BMD analysis was performed using the European Food Safety Authority web-tool integrated with the R-package PROAST version 66.90 developed by the Dutch National Institute for Public Health and the Environment (RIVM) as previously described (Shi et al. 2020b). Unlike the concentration–response curve itself, the accompanying confidence intervals of rac-methadone could not be assigned or distributed to the two enantiomers. Therefore, the BMDL_10_ values (lower 95% confidence limit of BMD_10_) were derived by dividing BMD values by 3 given that a reliable BMDL value should be at most three-fold lower than the corresponding BMD value (EPA 2012). BMDL_10_ values were defined for the average group and for the sensitive group using the CSAF calculated based on variations in multiple parameters, which allows the extrapolation of inter-individual kinetic variations in metabolic conversion and other parameters (body weight, the oral fraction absorbed and the fraction unbound in plasma) to variation in external toxic dose levels.

The Margin of safety (MOS) is an important concept in the safety evaluation of drugs. In the classic approach, the MOS for the drug safety in pharmaceutical industry is the ratio of the lethal dose or toxic dose to 1% of the population (LD_1_ or TD_1_) to the effective dose to 99% of the population (ED_99_) and would require in vivo data representative for the population. To obtain insight in the influence of inter-individual variation on the toxicological profile for the risk–benefits of a compound earlier in the drug developmental process BMDLs derived using the presented in vitro–in silico approach can be integrated in the MOS approach. In this example, the predicted BMDL_10_ of the enantiomers for the sensitive population (99th percentile of the *C*_max_ in the heart venous blood) was chosen as an alternative to the TD_1_ and the therapeutic dose of rac-methadone served as ED_99_, because information on therapeutic doses of enantiomers is not available.

## Results

### Metabolic variation in the conversion of R- and S-methadone

#### In vitro incubation of 25 Caucasian and 25 Chinese individual liver microsomes

The conversion of R- and S-methadone toward R- and S-EDDP was measured in incubations with individual microsomes originating from the Caucasian and the Chinese population. The concentration-dependent increase in the formation of R- and S-EDDP followed Michaelis–Menten kinetics. The obtained apparent *V*_max_ and *K*_m_ values, and the calculated catalytic efficiencies derived from the data are summarized in Table 2 and individual results are shown in Table S9 in the supplementary materials 1. For the 25 Caucasian individuals, the differences between the individuals with the highest and lowest catalytic efficiency and the CV of the inter-individual differences in catalytic efficiency for R-methadone were 1.9- and 1.4-fold lower than the ones for S-methadone, respectively. The mean catalytic efficiency for R-methadone conversion to R-EDDP was 1.5-fold lower than that for S-methadone conversion. For the 25 Chinese individuals, a comparable variation in the metabolism of R- and S-methadone was observed for differences between the highest and lowest catalytic efficiency, the CV of the inter-individual differences in catalytic efficiency and the mean catalytic efficiency.

**Table 2:** Descriptive statistic of the kinetic constants V_max_, K_m_ and catalytic efficiencies for R-EDDP and S-EDDP formation by 25 Caucasian and 25 Chinese individual human liver microsomes

	Caucasian individuals						Chinese individuals					
	R-EDDP formation			S-EDDP formation			R-EDDP formation			S-EDDP formation		
	V_max_^a^	K_m_^b^	Catalytic efficiency^c^	V_max_^a^	K_m_^b^	Catalytic efficiency^c^	V_max_^a^	K_m_^b^	Catalytic efficiency^c^	V_max_^a^	K_m_^b^	Catalytic efficiency^c^
Mean (μ_x_)	0.40	155.1	2.87	0.34	111.3	4.27	0.064	127.7	0.55	0.046	115.6	0.47
SD^d^	0.324	42.8	2.64	0.319	44.2	5.54	0.031	42.4	0.35	0.021	63.6	0.28
CV_x_ %^e^	80.3	27.6	92.1	93.8	40	130	48.5	33.2	64.3	46.5	55	59.8
Fold-differences^f^	13	3	19	17	4	37	11	4	10	11	8	12

^a^nmol/min/mg liver microsomes

^b^µM

^c^V_max_/K_m_, µl/min/mg protein

^d^Standard deviation of kinetic constants

^e^Coefficients of variation, % (= SD/mean × 100)

^f^Highest/lowest values

Regarding the inter-ethnic variations in the metabolism of R-methadone, a 6.3-fold higher mean V_max_ value and a comparable mean K_m_ value were obtained for the Caucasian population compared to the Chinese population, resulting in a 5.2-fold higher mean catalytic efficiency in the Caucasian population than the Chinese population (Table 2). For the metabolism of S-methadone, the catalytic efficiency was ninefold higher in the Caucasian population than in the Chinese population, which is mainly due to a 7.4-fold higher mean *V*_max_ value since similar mean *K*_m_ values were observed in the Caucasian population compared to the Chinese population (Table 2). The CV of the catalytic efficiency for R- and S-methadone metabolism was, respectively, 1.4- and 2.2-fold higher in the Caucasian population than in the Chinese population.

#### Kinetic constants for R- and S-methadone conversion by rCYPs and variations in CYP abundances in the Caucasian and the Chinese population

Table 1 shows the in vitro kinetic constants *V*_max_ and *K*_m_ for the conversion of methadone enantiomers to EDDP enantiomers by the major CYPs as reported by Totah et al. (2007). The Table also presents the scaled catalytic efficiency for Caucasian and Chinese HLM for R- and S-methadone conversion taking into account the ISEF and the population specific CYP abundances to calculate *V*_max_ according to Eq. 2. Compared to the Chinese population, the Caucasian population has 3.2-fold higher, 2.5-fold higher and 1.3-fold lower abundances in CYP2B6, CYP2C19 and CYP3A4, respectively, with larger CVs.

### Sensitivity analysis and evaluation of the PBK model for R- and S-methadone

The sensitivity analysis shows that the SC of model parameters in the PBK model using the average individual HLM kinetic data were similar for R- and S-methadone at the two dose levels analysed (supplementary materials 1, Fig. S1). For both the Caucasian and the Chinese PBK model, the predicted steady-state *C*_max_ of R- or S-methadone in the heart venous blood was highly influenced by the following model parameters with the hierarchy of normalized SC values being the oral fraction absorbed (Fa) > body weight (BW) > liver metabolism-related parameters (VLc, MPPGL, *V*_max_, *K*_m_) > the absorption rate constant (ka). The renal clearance (RCL) was more influential in the Chinese model with a normalized SC value comparable to those of liver metabolism-related parameters. The results of the sensitivity analysis performed for the PBK model using rCYPs kinetic data were similar to the results obtained in the PBK model using HLM kinetic data (data not shown).

**Fig. 2:**
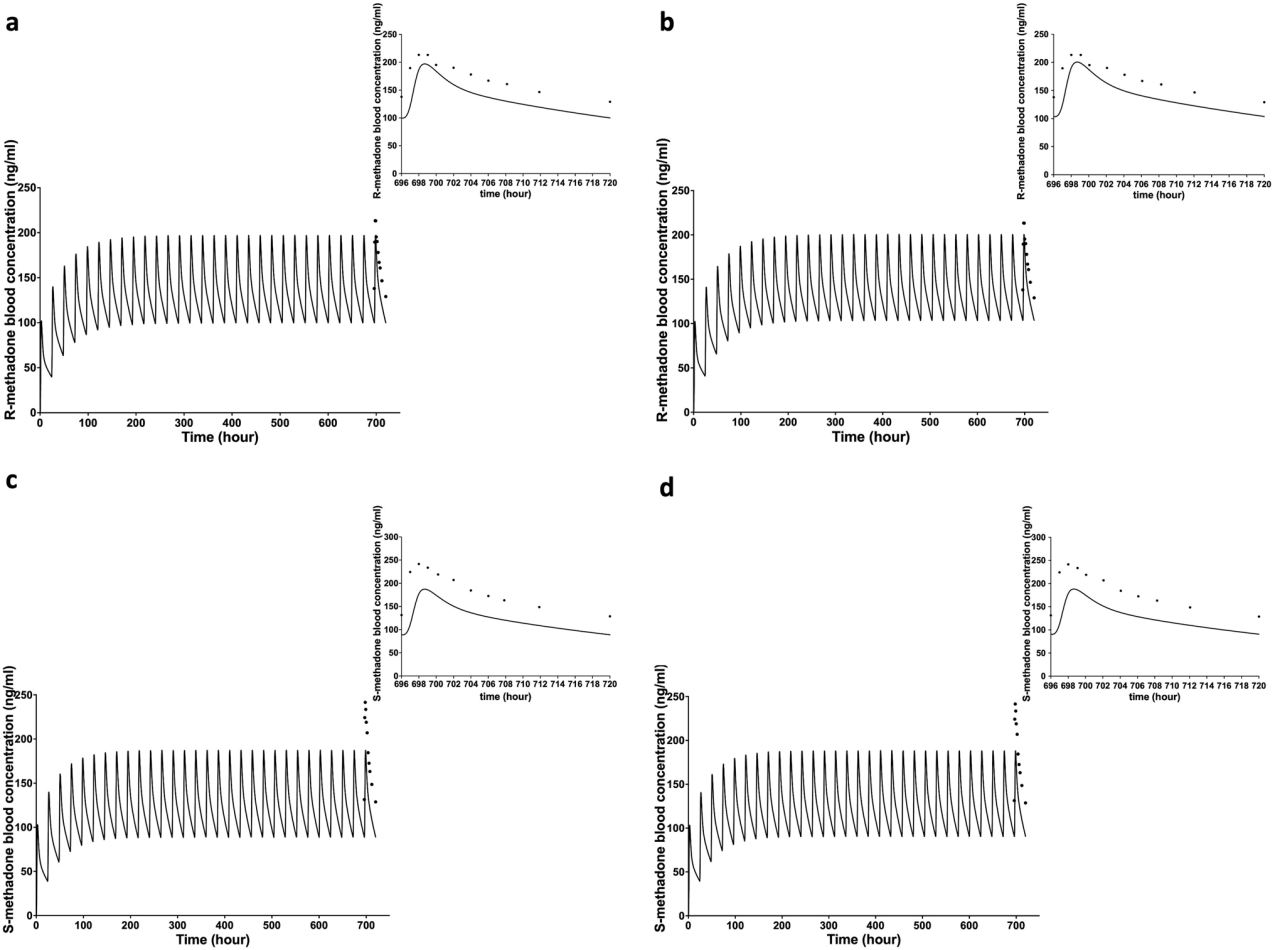
Blood concentration–time curves of R-methadone (**a**, **b**) and S-methadone (**c**, **d**) in human predicted with the PBK model (lines) and published in vivo data (dots) (Liu et al. 2007) after a repeated oral rac-methadone dose of 100 mg/day for 30 days. **a** and **c** present predictions obtained from the model using HLM kinetic data and **b** and **d** present predictions obtained from the model using rCYPs kinetic data. The top right insert is the predicted blood concentration of R- and S-methadone (lines) and in vivo data (dots) during the last 24 h upon the oral exposure

The developed PBK models using the average individual HLM kinetic data or rCYPs kinetic data for R- and S-methadone were evaluated against reported in vivo human data. Figure 2 reveals that for both models the predicted blood concentrations of R- and S-methadone during the last 24 h upon a repeated oral rac-methadone dose of 100 mg/day for 30 days, adequately matched with the corresponding in vivo data for Caucasian subjects (Liu et al. 2007). A similar comparison was obtained between predictions and in vivo human data from other studies (Foster et al. 2000; Garimella et al. 2015) as shown in Fig. S2 and S3 in the supplementary materials 1. Compared to data from three in vivo studies, when using the HLM kinetic data and rCYPs kinetic data, the prediction of kinetic values of R-methadone showed a 0.79- to 1.06-fold difference in steady-state *C*_max_ in venous blood and a 0.67-to 0.91-fold difference in AUC values. In case of S-methadone, the prediction showed a 0.95- to 1.36-fold difference in steady-state *C*_max_ in venous blood and a 0.75-to 1.08-fold difference in AUC values (Table S6 and S7 in the supplementary materials 1).

### Prediction of inter-individual and inter-ethnic variations applying individual PBK models and PBK modelling combined with Monte Carlo simulations

**Fig. 3:**
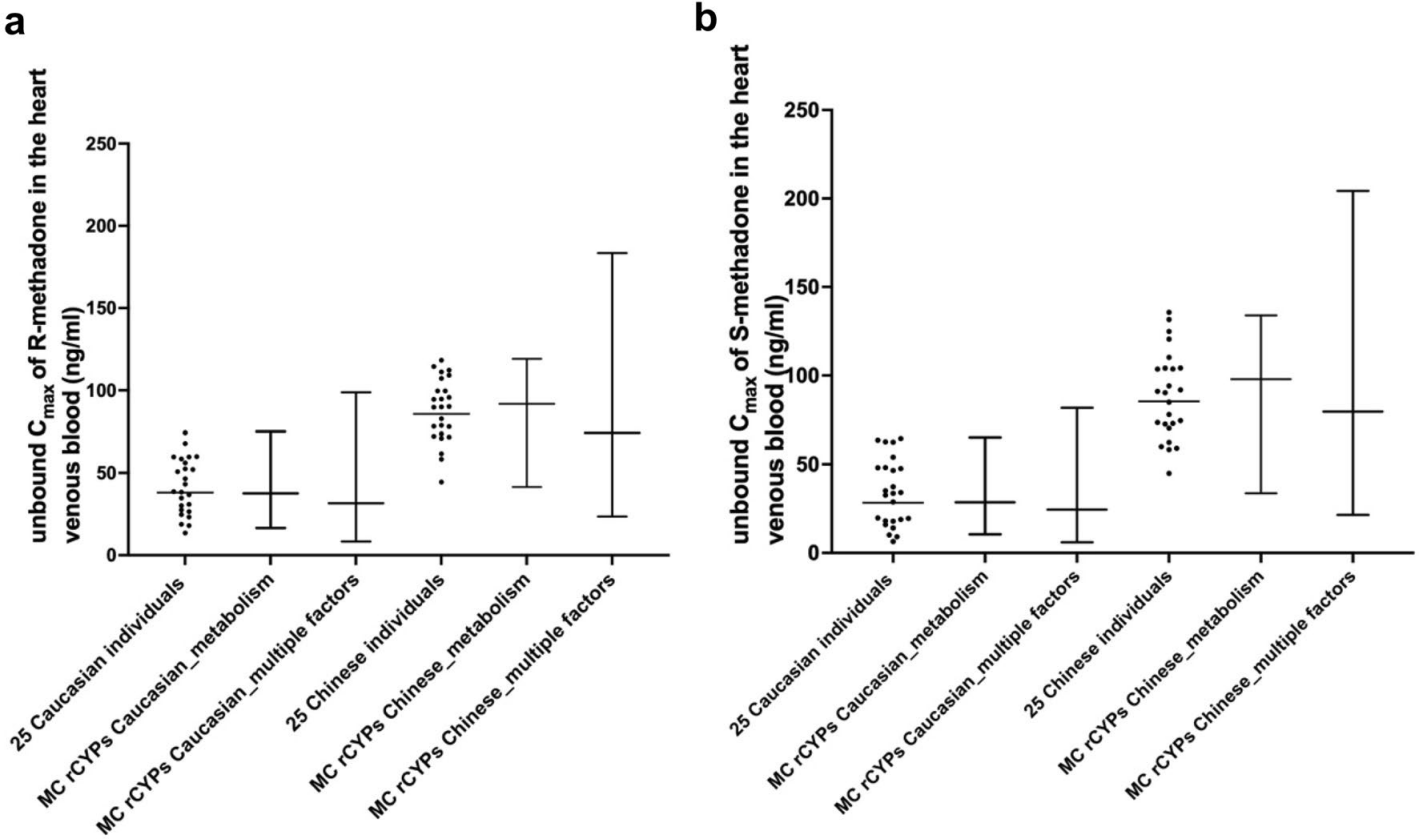
Distribution of predicted unbound C_max_ of R- (**a**) and S-methadone (**b**) in the heart venous blood at steady-state after a repeated oral methadone enantiomer dose of 30 mg/day for 30 days in the Caucasian and the Chinese population. The scatter plots represent the predictions obtained using individual PBK models. Whisker plots represent the predictions obtained by the Monte Carlo (MC) simulation considering the variations in metabolism only, and variations in multiple factors. The whiskers represent the 1st and 99th percentile of defined populations

The differences in the distribution of the predicted unbound *C*_max_ of R- and S-methadone in the heart venous blood among 25 Caucasian and 25 Chinese individuals is shown in Fig. 3. In the 25 Caucasian individuals, the geometric CV of the predicted unbound *C*_max_ of R-methadone in the heart venous blood was 1.6-fold lower than that of S-methadone, while in the 25 Chinese individuals, this value was 1.2-fold lower for R-methadone compared to S-methadone. For the inter-ethnic variations, the GM of predicted unbound *C*_max_ of R- and S-methadone in 25 Caucasian individuals was 2.2- and 3-fold lower than those in 25 Chinese individuals, respectively, and the geometric CVs observed in Caucasian individuals were 2.1 and 2.8-fold higher than those observed in Chinese individuals for R- and S-methadone, respectively.

The inter-individual and inter-ethnic differences in unbound *C*_max_ of R- and S-methadone in the heart venous blood predicted with the Monte Carlo simulations using variation in CYP abundances are shown in Fig. 3. Both for the Caucasian and the Chinese population, the geometric CVs and the differences between highest and lowest predicted unbound *C*_max_ of R- and S-methadone were comparable to those obtained from individual PBK models, except that the geometric CVs of predicted unbound *C*_max_ of S-methadone in the Caucasian population was 1.8-fold lower than the results obtained from the individual Caucasian PBK models. For the Caucasian population, the GM of unbound *C*_max_ of the two enantiomers were comparable with the GM values predicted using the individual PBK models and for the Chinese population the GM of predicted unbound *C*_max_ of R- and S-methadone were both 1.1-fold higher than when using individual PBK models. Furthermore, the inter-ethnic variations in GM and geometric CVs of predicted unbound *C*_max_ of the two enantiomers differ less than two-fold from the ones obtained using individual PBK models. Detailed predictions are shown in Table S10 in the supplementary materials 1.

Figure 3 also shows the inter-individual and inter-ethnic differences in unbound *C*_max_ of R- and S-methadone in the heart venous blood when considering variations in body weight, fraction absorbed and fraction unbound in plasma together with variation in CYP abundance. Both for the Caucasian and the Chinese population, the GM values of unbound *C*_max_ of R- and S-methadone were 1.2-fold lower than the values predicted considering variations in the metabolism only while the geometric CVs and the differences between highest and lowest predicted unbound *C*_max_ of R- and S-methadone were 1.5- to 2.7-fold higher than the results obtained from the prediction considering variations in the metabolism only. Detailed predictions are shown in Table S11 in the supplementary materials 1.

### Derivation of CSAF

**Fig. 4:**
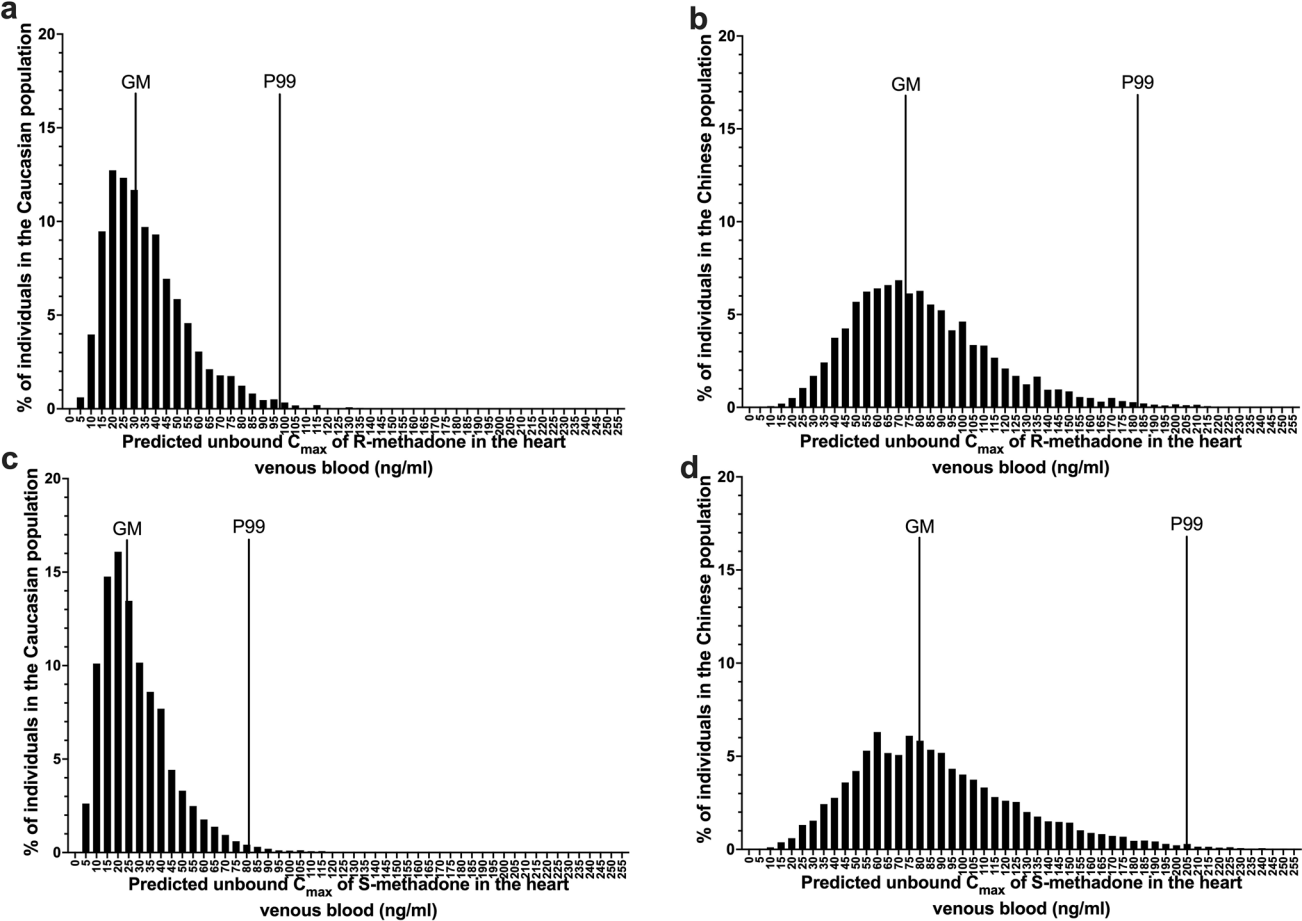
Frequency distribution for unbound C_max_ of R-methadone (**a**, **b**) and S-methadone (**c**, **d**) in the heart venous blood at steady-state after a repeated oral methadone enantiomer dose of 30 mg/day for 30 days in Caucasian (**a,** **c**) and Chinese (**b**, **d**) individuals by the Monte Carlo simulation considering variations in multiple factors. The GM and P99 represent the geometric mean and the 99th percentile of the distribution

CSAF values calculated for the Caucasian population, the Chinese population and the two populations combined are shown in Table 3. When considering variation in methadone metabolism only, the CSAFs for the Chinese were 1.4 to 1.6-fold lower than the Caucasian CSAFs, indicating a smaller inter-individual variation in the Chinese compared to the Caucasian population. When considering the variation in body weight, fraction absorbed and fraction unbound in plasma in addition to the variation in methadone metabolism, all CSAF values increased 1.3- to 1.9-fold compared to the CSAFs derived based on only metabolic variations. The frequency distribution of the unbound *C*_max_ of methadone enantiomers in the heart venous blood, considering variation in multiple parameters is shown in Fig. 4.

**Table 3:** CSAFs of R- and S-methadone for the Caucasian population, the Chinese population and the two populations combined, in each scenario of Monte Carlo simulation taking into account variations in metabolism, bodyweight, oral fraction absorbed and fraction unbound in plasma

	CSAFs at 95th percentile			CSAFs at 99th percentile		
	Caucasian population^a^	Chinese population^a^	Two populations combined^b^	Caucasian population^a^	Chinese population^a^	Two populations combined^b^
R-methadone	2.4 (1.7)	1.9 (1.2)	4.5 (3.0)	3.1 (2.0)	2.5 (1.3)	5.8 (3.2)
S-methadone	2.5 (1.9)	2.1 (1.3)	6.7 (4.5)	3.3 (2.3)	2.6 (1.4)	8.3 (4.7)

Values between brackets are the CSAFs derived on the metabolic variation only

^a^Obtained by dividing the 95th or 99th percentile of the unbound C_max_ in heart venous blood by the GM of the unbound C_max_ in heart venous blood in each population

^b^Obtained by dividing the 95th or 99th percentile of the unbound C_max_ in heart venous blood in the Chinese population by the GM of the unbound C_max_ in heart venous blood in the Caucasian population

### PBK modelling-based reverse dosimetry and BMD analysis of in vivocardiotoxicity of methadone

PBK modelling-based reverse dosimetry was applied to further investigate the consequences of obtained inter-individual and inter-ethnic kinetic variations for the predicted in vivo cardiotoxicity of R- and S-methadone. To this end, first the in vitro derived cardiotoxicity of rac-methadone obtained in the hiPSC-CM multi-electrode array assay (Shi et al. 2020a) was transformed to cardiotoxicity data for the individual R- and S-enantiomer, based on the reported hERG channel inhibition potencies of the two enantiomers (Eap et al. 2007). The thus obtained in vitro concentration–response curves of R- and S-methadone are shown in Fig. S4 in the supplementary materials 1.

**Fig. 5:**
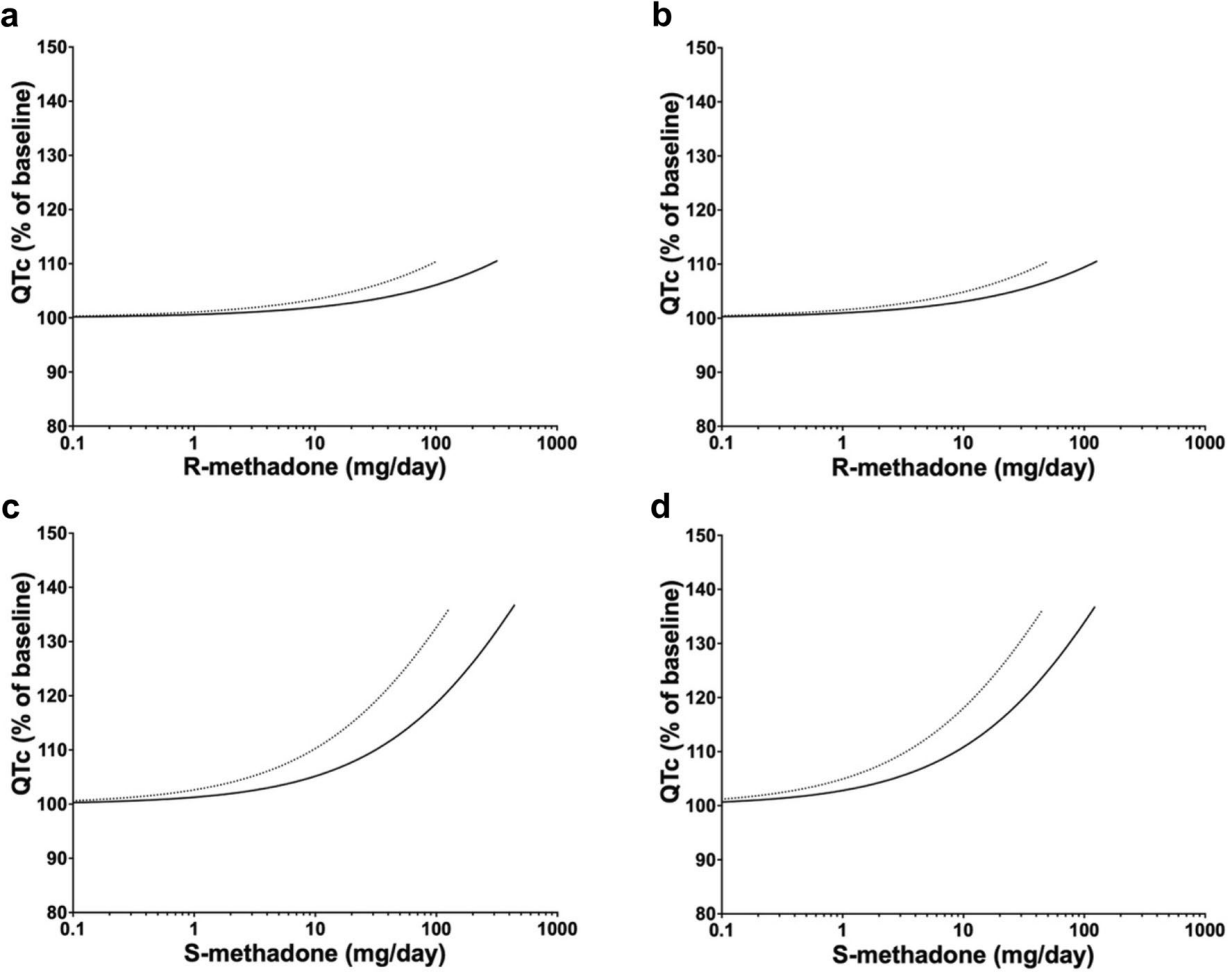
Predicted dose–response curves for the cardiotoxicity of R-methadone (**a**, **b**) and S-(**c**, **d**) methadone in the average (solid lines) and the sensitive population (99th percentile of predicted C_max_ in heart venous blood) (dotted lines) of Caucasian (**a**, **c**) and Chinese (**b**, **d**) population. The dose–response curves for sensitive Caucasian and Chinese populations were obtained by applying the respective CSAFs (calculated based on variations in metabolism, bodyweight, oral fraction absorbed and fraction unbound in plasma.) to the dose–response curves of the average populations

After applying the reverse dosimetry, the in vivo dose–response curves for R- and S-methadone for the average and sensitive population for the both the Caucasian and Chinese population (Fig. 5) indicate a larger variation in both R- and S-methadone-induced human cardiotoxicity for the Caucasian population compared to the Chinese population. Table 4 shows that the BMDL_10_ values of R- and S-methadone for the average Caucasian were respective 3.1- and 3.4-fold higher than the BMDL_10_ values of the sensitive Caucasians. For the average Chinese the BMDL_10_ values of R- and S-methadone were respective 2.5- and 2.7-fold higher than the BMDL_10_ values for the sensitive Chinese. BMDL_10_ values of S-methadone for the average and the sensitive Caucasians were, respectively, 3.7- and 2.9-fold higher than the corresponding values for the Chinese, indicating that the Caucasians may be less sensitive to methadone-induced cardiotoxicity than the Chinese (Table 4). The predicted MOS values are summarized in Table 5. For both enantiomers, the MOS values for the Caucasians were 2.1- to 2.9-fold higher than the MOS values for the Chinese. The MOS values for R-methadone were 5.9- to 8.3-fold higher than the ones for S-methadone.

**Table 4:** The predicted BMDL_10_ values for the average and the sensitive (99th percentile of predicted C_max_ in heart venous blood) of the Caucasian and Chinese population obtained by the CSAFs derived from the Monte Carlo simulation considering variations in metabolism, bodyweight, oral fraction absorbed and fraction unbound in plasma

	Caucasian population		Chinese population	
	R-methadone	S-methadone	R-methadone	S-methadone
BMDL_10_ (mg/day) for the average population	99.6	18.7	39.5	5.1
BMDL_10_ (mg/day) for sensitive population	32.4	5.5	15.8	1.9

**Table 5:** Summary of Margin of Safety (MOS) values for R- and S-methadone for the Caucasian and Chinese population

Enantiomer	Stage dosing	Effective dose (rac-methadone mg/day)	Toxic dose^a^		Margin of Safety (MOS)^b^	
			(enantiomer mg/day)			
			Caucasian	Chinese	Caucasian	Chinese
R-methadone	Initial	10	32.4	15.8	3.2	1.6
	Maintenance	60	32.4	15.8	0.5	0.3
S-methadone	Initial	10	5.5	1.9	0.6	0.2
	Maintenance	60	5.5	1.9	0.09	0.03

The MOS is defined as the ratio between the predicted BMDL_10_ of the enantiomers for the sensitive population (99th percentile of the C_max_ in the heart venous blood) and the therapeutic dose of rac-methadone

^a^BMDL_10_ values for the sensitive population (99th percentile of C_max_ in heart venous blood) were used as toxic dose for 1% population (TD_1_)

^b^Obtained by dividing TD_1_ by effective dose

## Discussion

The aim of present study was to apply a NAM approach that combines in vitro data, PBK modelling and Monte Carlo simulations to predict inter-ethnic and inter-individual kinetic variations in the R- and S-methadone, and to elucidate consequences of these variations for the sensitivity towards the cardiotoxicity of both enantiomers. CSAFs were derived to quantitatively reflect the inter-ethnic and inter-individual variation in kinetics and used to derive dose–response curves for the sensitive individual in the population. Applying the presented NAM in drug safety evaluation contributes to the 3Rs, since the approach reduces the need for animal studies on cardiotoxicity.

In the current paper, two sources of metabolic data were integrated in the PBK model to define the inter-individual differences in the metabolism of R- and S-methadone. First, the formation of both enantiomers was determined with 25 Caucasian and 25 Chinese individual HLMs and kinetic constants obtained were used to define 50 individual PBK models. The catalytic efficiencies for the metabolism of both enantiomers were 5- to 9-fold higher in incubations with the Caucasian HLMs compared to Chinese HLMs, and, as a result, the PBK model predictions revealed a notable inter-ethnic difference in the predicted venous blood concentrations. The observed differences may be explained by the inter-ethnic differences in the distribution of functional alleles and in the abundance of CYP2B6 and CYP3A. Zhou et al. (2017) found that the overall frequency of CYP2B6 alleles, associated with higher catalytic activity, was two-fold higher in Europeans compared to the East Asians. Especially, two key allelic mutations (516G > T and 785A > G), known to increase the in vitro catalytic efficiency for conversion of 7-ethoxy-4-trifluoromethylcoumarin (Jinno et al. 2003) and cyclophosphamide (Xie et al. 2003), have higher frequencies in the Caucasian population compared to the Chinese population (Guan et al. 2006). The CYP3A4*20 allele, reported to result in inactived catalytic activity (Zhou et al. 2017), showed a 3.7-fold lower prevalence in the Caucasian population compared to the East Asians (McGraw and Waller 2012) and the CYP3A4*22 allele, which decreases the activity and protein expression, had a higher frequency in the Asian population (0.043) compared to the Caucasians (0.008–0.025) (Zanger and Schwab 2013). Furthermore, the reported hepatic abundances of CYP2B6 and CYP3A4 (EM) were up to three-fold higher in Caucasians compared to the Chinese (Barter et al. 2013).

Another factor contributing to the ethnic differences in the catalytic efficiency and predicted kinetics could be the content of cytochrome b5 (Cyt b5) in the individual HLM. It is important to note that Cyt b5 plays an important role in the CYP-mediated reactions where Cyt b5 may provide the second electron to the monooxygenase cycle (Kandel and Lampe 2014). Many studies indicated that Cyt b5 stimulated the catalytic activity of CYP2B6 and CYP3A4. Yamazaki et al. (1996) demonstrated that the catalytic efficiency for testosterone (CYP3A4 substrate) in the presence of Cyt b5 was four-fold higher than the one without Cyt b5. Zhang et al. (2015a) found a positive correlation between the Cyt b5 content and the catalytic efficiency (and *V*_max_) for CYP2B6 in Chinese HLM, which was in agreement with the positive correlation we found between the Cyt b5 content and the catalytic efficiency (and V_max_) for both enantiomers in 25 Caucasian and 25 Chinese individual HLMs (Fig. S5 in the supplementary materials 1), suggesting that Cyt b5 may influence the catalytic efficiency for methadone enantiomers. The mean Cyt b5 content in HLMs used in the current study was 455 pmol/mg for Caucasians HLMs and 199 pmol/mg for Chinese. Values that are comparable to reported values being 660 pmol/mg for Caucasians (Corning 2014) and 270 pmol/mg for Chinese (Zhang et al. 2015a). Thus, the lower conversion of methadone by the Chinese HLM may in part also be ascribed to the lower Cyt b5 content in these HLM.

Our results reveal that Caucasians had in general two-fold higher CVs for the catalytic efficiency and predicted venous blood concentrations for both enantiomers than Chinese. This can be partly explained by the fact that the CV of CYP3A4 abundance is three-fold higher for Caucasians compared to Chinese (Achour et al. 2014; Shu et al. 2000), and that the allelic variants of CYP2B6 and CYP3A4 appeared to be more frequent in Caucasian than in Asian (Li and Bluth 2011). The latter fact may also explain the larger variations in the kinetics of S-methadone within the Caucasian population observed in the current study, given that CYP2B6 shows stereoselectivity towards to metabolism of S-methadone (Chang et al. 2011).

The reported CYP abundances combined with kinetic data for the respective rCYPs were used as the second source to describe inter-individual variation in metabolism of methadone enantiomers using a PBK model integrated with Monte Carlo simulations. Generally, the predicted GM of *C*_max_ in the heart venous blood and the corresponding CV values were comparable with the results obtained from the individual PBK models, especially for the Caucasian population. For the Chinese population, the predicted GM of *C*_max_ in the heart venous blood for the two enantiomers showed a 1.2-fold differences between the two approaches. The reasons underlying this observation may be related to the fact that the ISEF values used for the Chinese models were derived using *f*_m,CYP_ obtained from Caucasian microsomes due to the absence of Chinese microsomal data. The derivation of ISEFs was reported to vary among studies and to be dependent on the accessory proteins (Chen et al. 2011; Crewe et al. 2011). Crewe et al. (2011) demonstrated that ISEFs for CYP2C9 differed up to ten-fold between rCYP systems with and without Cyt b5, indicating that ISEFs were sensitive to the differences in Cyt b5 and the CYP450/Cyt b5 ratio can be used to indicate the influences of Cyt b5 variations on ISEFs. In the current study, the CYP450/Cyt b5 ratio of 25 Caucasian HLM is 1 while the ratio for 25 Chinese HLM was much higher amounting to around 10 (Table S1, in the supplementary materials 1). Given that both the Cyt b5 content and CYP450/Cyt b5 ratio differed between the two populations, the ISEFs determined based on Caucasian microsomal kinetics may not completely capture the discrepancy between rCYP system and HLM system for the Chinese population. Further studies on Chinese specific ISEFs are needed to improve the prediction. Moreover, together with reporting rCYPs catalytic data, assay specific ISEF values should ideally also be derived and reported, since the absence of these ISEFs hamper the use of reported rCYPs data, for example for QIVIVE.

Altogether, comparing the results obtained from the individual PBK models and the Monte Carlo prediction (considering variations in the metabolism only) indicates that both approaches similarly predict the inter-individual and inter-ethnic variations in the kinetics of R-methadone and to a somewhat lesser extent that of S-methadone. This implies that both groups of 25 Caucasian and 25 Chinese individuals were able to represent the inter-ethnic kinetic variations in CYP metabolism between Caucasians and Chinese on a population level. When including the variation in body weight, fraction absorbed, and fraction unbound in plasma in addition to variation in parameters for methadone metabolism, the CSAF values can be compared to the default safety factor of 3.16 for kinetic differences used in chemical risk assessments (IPCS 2005). A limited inter-individual kinetic variation of less than 3.16 in each individual population for both enantiomers was observed, except for protection of the 99th percentile of the Caucasian population in the case of exposure to S-methadone. For the combined (Chinese plus Caucasian) population, the obtained CSAFs for S- and R-methadone were higher than the default of 3.16, namely, 6.7 and 8.3 for the 95th and 99th percentile of the population for S-methadone and 4.5 and 5.8 for the 95th and 99th percentile of the population for R-methadone, respectively. The CSAFs considering variation in methadone metabolism only, are lower than 3.16 for the individual populations as well as the combined populations, except for the CSAFs for S-methadone that was somewhat higher that 3.16 in the combined population. Overall, considering the variation in multiple parameters instead of metabolism only, provides higher and thus more protective CSAFs.

For both enantiomers, the MOSs were 2- to 3-fold higher for the Caucasians compared to the Chinese, indicating that, based on the kinetic differences observed, the Chinese population may be at extra risk towards methadone-induced cardiotoxicity. Often individuals are exposed to rac-methadone. We previously derived the BMDL_10_ of 24 mg/kg for rac-methadone for the average Caucasian (Shi et al. 2020a). After applying the same approach used for R- and S- methadone for rac-methadone, the MOS values of rac-methadone obtained were 4- to 9-fold lower than the MOS values of R-methadone for the Caucasians. This indicates that administering only R-methadone might decrease the risk of methadone-induced cardiotoxicity, which is in agreement with the study of Ansermot et al. (2010) where the replacement of rac-methadone by R-methadone was shown to reduce the prolonged QTc interval in opioid addiction patients. Thus, for therapeutic use, administering R-methadone (and not rac-methadone) is highly recommended.

In the current study, the inter-ethnic and inter-individual variation in methadone-induced cardiotoxicity were assessed using the variability in physiological and kinetic parameters, which could to some extent reflect the overall variability in the populations given the significant role of variation in methadone blood concentrations in the variability in dose–response relationship in the clinical settings (Eap et al. 2002; Li et al. 2008). HiPSC-CM derived from different donors have been demonstrated as a tool to study the inter-individual variability in drug-induced cardiotoxic effects (Burnett et al. 2021). To what extent a chemical shows inter-individual variation in cardiotoxicity may be chemical-specific and also dependent on the type of dynamic endpoint(s) studied (e.g. QT prolongation, beating rate, peak amplitude and cell viability) (Blanchette et al. 2020; Burnett et al. 2019; Grimm et al. 2018). Due to lack of such data on methadone, toxicodynamic variations were not included in our approach, but future data on potential inter-ethnic and inter-individual variation in toxicodynamics could be included in future studies on inter-individual variation in methadone-induced cardiotoxicity and used to further refine the CSAF.

In conclusion, we demonstrated that integrating in vitro cardiotoxicity data, PBK modelling and Monte Carlo simulation is a powerful approach to predict the influence of inter-ethnic and inter-individual kinetic variations for the sensitivity towards R- and S-methadone-induced cardiotoxicity. PBK models based on either HLM kinetics or rCYPs kinetics similarly predicted the inter-ethnic and inter-individual kinetic variations for the methadone enantiomers. Our findings also revealed the importance of the scaling factors used when rCYP systems are applied. Furthermore, based on the kinetic differences, Chinese individuals were predicted to be more sensitive towards methadone-induced cardiotoxicity making it even more important to replace rac-methadone by R-methadone to decrease the risk of methadone-induced cardiotoxicity in the clinical setting. Altogether, the present study shows that this PBK modeling-based NAM approach combining in vitro data and in silico modelling is promising to predict the role of kinetics in inter-ethnic and inter-individual variation in cardiotoxicity, which can be used to refine the cardiac safety evaluation and risk assessment of chemicals.

## Supplementary Information

Below is the link to the electronic supplementary material.

Supplementary file1 (PDF 861 kb)

## Data Availability

Supplementary materials (model code and supplementary figures).

## Abbreviations

ADME: Absorption, distribution, metabolism and excretion
AUC: Area under the curve
BMD: Benchmark dose
BMD_10_: Benchmark dose resulting in 10% effect
BMDL_10_: Lower 95% confidence limit of BMD resulting in 10% effect
ED_99_: Effective dose to 99% of the population
EDDP: 2-Ethylidene-1,5-dimethyl-3,3-diphenylpyrrolidine
C_max_: Maximum blood concentration
CVs: Coefficients of variation
Cyt b5: Cytochrome b5
CSAFs: Chemical-specific adjustment factors
FPDc: Field potential duration corrected for beat rate
GM: Geometric mean
hERG: Human ether-à-go-go-related gene
hiPSC-CM: Human-induced pluripotent stem cell-derived cardiomyocytes
HLMs: Human liver microsomes
ISEF: Inter-system extrapolation factor
LD_1_: Lethal dose to 1% of the population
MOS: Margin of safety
MPPGL: Microsomal protein per gram of liver
NAM: New approach methodology
QIVIVE: Quantitative in vitro to in vivo extrapolation
rac-methadone: Racemic methadone
rCYPs: Recombinant cytochrome P450 enzymes
SC: Sensitivity coefficient
TD_1_: Toxic dose to 1% of the population

## Code availability

Model code available in supplementary materials.
