# Supplementary material for: In vitro–in silico-based prediction of inter-individual and inter-ethnic variations in the dose-dependent cardiotoxicity of R- and S-methadone in humans
Source: Arch Toxicol. 2022 May 23;96(8):2361–80. doi: 10.1007/s00204-022-03309-y (PMC9217890; doi:10.1007/s00204-022-03309-y)
Supplement: Supplementary file 1 — Supplementary file1 (PDF 861 kb) [file 204_2022_3309_MOESM1_ESM.pdf]

In vitro-in silico-based prediction of inter-individual and inter-ethnic variations in the dose-dependent cardiotoxicity of R- and S-methadone in humans

Miaoying Shi [1,2], Yumeng Dong [1], Hans Bouwmeester [1], Ivonne M. C. M. Rietjens [1], Marije Strikwold [3]

[1] Division of Toxicology, Wageningen University, Stippeneng 4, 6708 WE Wageningen, The Netherlands

[2] NHC Key Laboratory of Food Safety Risk Assessment, Chinese Academy of Medical Sciences Research Unit (No. 2019RU014), China National Center for Food Safety Risk Assessment, Beijing 100021, China

[3] Van Hall Larenstein University of Applied Sciences, 8901 BV Leeuwarden, The Netherlands

ORCID: Miaoying Shi 0000-0002-9159-1461

Corresponding author:
Miaoying Shi
Division of Toxicology, Wageningen University
Stippeneng 4, 6708 WE Wageningen, The Netherlands
Tel: +31-317 483334
Fax: +31-317 484931
Email: smy920@outlook.com

Supplementary materials 1

Table S1 Information of the human liver microsome donors

Caucasian			Chinese		
Code [a]	Cytochrome b5 (nmol/mg protein)	Cytochrome P450 (nmol/mg protein)	Code [a]	Cytochrome b5 (nmol/mg protein)	Cytochrome P450 (nmol/mg protein)
#Ca1	0.414	0.234	#Ch1	0.220	1.65
#Ca2	0.365	0.249	#Ch2	0.195	1.45
#Ca3	-	-	#Ch3	0.265	1.85
#Ca4	0.479	0.344	#Ch4	0.135	1.25
#Ca5	0.441	0.306	#Ch5	0.195	1.93
#Ca6	0.586	0.344	#Ch6	0.180	1.45
#Ca7	0.563	0.366	#Ch7	0.175	1.78
#Ca8	0.517	0.149	#Ch8	0.205	1.95
#Ca9	0.409	0.201	#Ch9	0.170	1.32
#Ca10	0.317	0.239	#Ch10	0.265	1.91
#Ca11	0.477	0.238	#Ch11	0.305	2.15
#Ca12	0.443	0.129	#Ch12	0.185	1.15
#Ca13	0.548	0.577	#Ch13	0.215	2.25
#Ca14	0.364	0.629	#Ch14	0.135	1.05
#Ca15	0.688	0.545	#Ch15	0.265	2.15
#Ca16	0.484	0.206	#Ch16	0.195	1.81
#Ca17	0.375	0.205	#Ch17	0.150	1.09
#Ca18	0.432	0.174	#Ch18	0.175	1.91
#Ca19	0.369	0.219	#Ch19	0.225	1.90
#Ca20	0.294	0.185	#Ch20	0.190	1.85
#Ca21	0.646	0.328	#Ch21	0.185	1.66
#Ca22	0.379	0.268	#Ch22	0.215	2.15
#Ca23	0.299	0.235	#Ch23	0.205	2.05
#Ca24	0.355	0.280	#Ch24	0.205	2.00
#Ca25	0.429	0.468	#Ch25	0.115	1.60

-, data are not available. [a] Liver microsomal fraction code from supplier.

Table S2 In vitro intrinsic clearance in rCYPs and HLM system and ISEF values for R-and S-methadone of three CYPs

		HLM		rCYP				
	CYP	$CL_{int, HLM}$ (µl/min/mg protein) (Caucasian/Chinese) [a]	$CL_{int, CYP in HLM}$ (µl/min/mg protein) (Caucasian/Chinese) [b]	$V_{max, CYP}$ (pmol/min/pmol CYP) [c]	$K_{m, CYP}$ (µM) [c]	$CL_{int, CYP}$ (µl/min/pmol CYP) [d]	Mean CYP abundance (pmol/mg protein) (Caucasian/Chinese) [e]	ISEF (Caucasian/Chinese)
R-methadone	2B6	2.6/0.5	1.16/0.22	36	60	0.6	15.4/5.1	0.13/0.072
	2C19		0.24/0.05	22	97	0.23	11/4.4	0.1/0.047
	3A4		1.21/0.23	43	137	0.31	93/120	0.04/0.0062
S-methadone	2B6	3.1/0.4	1.81/0.24	15	16	0.94	15.4/5.1	0.13/0.049
	C19		0.27/0.04	8	125	0.06	11/4.4	0.39/0.13
	3A4		0.97/0.13	46	149	0.31	93/120	0.03/0.0034

[a] calculated as V_{max}/K_m, using the HLM data obtained in the current study, [b] calculated as $Cl_{int,HLM} * f_{m,CYP}$, [c] reported in Totah et al. (2007), [d] calculated as $V_{max, CYP}/K_{m, CYP}$, [e] obtained from literature as reported in Table 5.

Table S3 Fraction metabolized of three CYPs to the metabolism of R-and S-methadone in HLM

CYP	Contribution of individual CYP to total metabolic clearance in HLM [a]		$f_{m,\ CYP}$ [b]	
	R-methadone	S-methadone	R-methadone	S-methadone
2B6	43.4%	69.2%	0.44	0.59
2C19	9.1%	10.4%	0.09	0.09
3A4	45.3%	37.1%	0.46	0.32

[a] average of results from five HLM samples as reported in Totah et al. (2008), [b] proportionally scaled the reported contribution of each CYP enabling the sum of $f_{m,CYP}$ equal to 1.

Table S4 Physiological parameters used in the PBK models for the Caucasian and the Chinese populations

Parameters	Symbol in model code	Caucasian [a]	Chinese [b]
Body weight (kg)	BW	70	58.5
Tissue volume (% body weight)			
Liver	VLc	2.57	2.3
Fat	VFc	21.4	18.4
Lung	VLuc	0.76	1.9
Arterial blood	VAc	1.98	1.98 [c]
Venous blood	VVc	5.93	5.93 [c]
Kidney	VKc	0.4	0.47
Heart	VHc	0.47	0.53
Slowly perfused tissue	VSc	53.18	57.35

Rapidly perfused tissue	VRc	5.2	3.99
Cardiac output (l/h)	Qc	347.9	327.0
Blood flow to tissue (% cardiac output)			
Liver	QLc	22.7	26.25
Fat	QFc	5.2	6.75
Kidney	QKc	17.5	17.5
Heart	QHc	4	4 [c]
Slowly perfused tissue	QSc	18.8	24.5
Rapidly perfused tissue	QRc	31.8	21

[a] reported in Brown et al. (1997), [b] reported in NHFPC (2007a, b, 2014), [c] data are not available for the Chinese population and were assumed to be the same as the ones for the Caucasian, given that the parameters are not influential on the model outcome, as can be seen in the result of sensitivity analysis.

Table S5 Physicochemical parameters used in the PBK model for R- and S- methadone

Parameters	Symbol in model code	R-methadone	S-methadone
Tissue: blood partition coefficients [a]			
Liver	PL	12.53	11.99
Fat	PF	3.33	2.54
Lung	PLu	1.77	1.71
Kidney	PK	7.6	7.29
Heart	PH	4.93	4.73

Slowly perfused tissue	PS	7.71	7.39
Rapidly perfused tissue	PR	12.53	11.99
Oral absorption rate constant (/h)	ka	0.59 [b]	0.59 [b]
Fraction absorbed	Fa	0.88 [c]	0.88 [c]
Renal clearance (l/h)	RCL	1.8 [c]	1.1 [c]

[a] obtained by dividing tissue: plasma partition coefficients by the corresponding BPr values, [b] reported in Foster et al. (2000), [c] reported in Ke et al. (2014).

Table S6 Summary of in vivo kinetic studies and evaluation of the PBK model predictions using HLM data for R- and S-methadone steady-state C_{max} in venous blood and AUC values based on the data derived from in vivo kinetic studies

Enantiomer	Body weight (kg)	Dose (mg/day) [a]	In vivo C_{max} (ng/ml) [b]	In vivo AUC (ng · h/ml) [b]	Predicted C_{max} (ng/ml)	Predicted AUC (ng · h/ml)	Ratio in vivo C_{max}/predicted C_{max}	Ratio in vivo AUC /predicted AUC	Reference
R-methadone	74	35	175.7	2439	165.8	2728	1.06	0.89	Foster et al. (2000)
	90	50	179.2	3010	200.6	3303	0.89	0.91	Liu et al. (2007)
	70	20	77.9	1099	99.2	1631	0.79	0.67	Garimella et al. (2015) [c]
S-methadone	74	35	212.1	2658	157.0	2512	1.35	1.06	Foster et al. (2000)
	90	50	200.9	2821	188.3	3003	1.07	0.94	Liu et al. (2007)
	70	20	89.5	1134	94.2	3138	0.95	0.75	Garimella et al. (2015) [c]

[a] free base form of R- or S-methadone, [b] obtained by multiplying reported plasma kinetics by the BPr values, [c] the body weight of subjects was set equal to the value used in the PBK model since body weight of study subjects was not reported.

Table S7 Summary of in vivo kinetic studies and evaluation of the PBK model predictions using rCYPs data for R- and S-methadone steady-state C_{max} in venous blood and AUC values based on the data derived from in vivo kinetic studies

Enantiomer	Body weight (kg)	Dose (mg/day) [a]	In vivo C_{max} (ng/ml) [b]	In vivo AUC (ng · h/ml) [b]	Predicted C_{max} (ng/ml)	Predicted AUC (ng · h/ml)	Ratio in vivo C_{max}/predicted C_{max}	Ratio in vivo AUC /predicted AUC	Reference
R-methadone	74	35	175.7	2439	162.4	2643	1.08	0.92	Foster et al. (2000)
	90	50	179.2	3010	197.1	3214	0.91	0.94	Liu et al. (2007)
	70	20	77.9	1099	96.5	1562	0.81	0.70	Garimella et al. (2015) [c]
S-methadone	74	35	212.1	2658	155.4	2467	1.36	1.08	Foster et al. (2000)
	90	50	200.9	2821	187.3	2969	1.07	0.95	Liu et al. (2007)
	70	20	89.5	1134	91.3	3026	0.97	0.78	Garimella et al. (2015) [c]

[a] free base form of R- or S-methadone, [b] obtained by multiplying reported plasma kinetics by the BPr values, [c] the body weight of subjects was set equal to the value used in the PBK model since body weight of study subjects was not reported.

Table S8 Summary of distribution parameters of hepatic CYP abundances

CYP	Caucasian					Chinese				
	Phenotype (frequency)	Mean abundance (μ_x, pmol/mg protein)	CV (%)	μ_ω [a]	σ_ω [b]	Phenotype (frequency)	Mean abundance (μ_x, pmol/mg protein)	CV (%)	μ_ω [a]	σ_ω [b]
2B6	EM (0.89) [c]	17 [c]	122 [c]	2.38	0.95	EM (0.95) [d]	5.3 [c]	198 [c]	0.87	1.26
	PM (0.11) [c]	6 [c]	200 [c]	0.99	1.27	PM (0.05) [d]	1.9 [c]	200 [c]	-0.16	1.27
2C19	General group	11 [e]	82 [e]	2.14	0.72	EM (0.87) [c]	4.4 [c]	39 [c]	1.41	0.38
3A4	General group	93 [e]	81 [e]	4.28	0.71	EM (1)[d]	120 [f]	33 [f]	4.74	0.32

EM, extensive metabolizer; PM, poor metabolizer; [a] the mean of the log-normal distribution $\mu_\omega=\ln[\mu_x /\mathrm{sqrt}\ (1+CV_x^2)]$ [b] the standard deviation of the log-normal $\sigma_\omega = \mathrm{sqrt}[\ln(1+CV_x^2)]$ distribution, [c] obtained from the Simcyp simulator V18 Release 1 (Certara), [d] based on Guan et al. (2006), [e] values were summarized by Achour et al. (2014) from different studies, [f] reported by Shu et al. (2000)

Table S9 Kinetic constants V_{max}, K_m and catalytic efficiencies for R-EDDP and S-EDDP formation by 25 Caucasian and 25 Chinese individual human liver microsomes

Caucasian individuals	R-EDDP formation			S-EDDP formation			Chinese individuals	R-EDDP formation			S-EDDP formation		
	V_{max} ± SEM [a]	K_m ± SEM [b]	Catalytic efficiency [c]	V_{max} ± SEM [a]	K_m ± SEM [b]	Catalytic efficiency [c]		V_{max} ± SEM [a]	K_m ± SEM [b]	Catalytic efficiency [c]	V_{max} ± SEM [a]	K_m ± SEM [b]	Catalytic efficiency [c]
#Ca1	0.136 ± 0.007	134.1 ± 19.9	1.02	0.078 ± 0.04	111.9 ± 17.9	0.70	#Ch1	0.071 ± 0.007	181.9 ± 49.2	0.39	0.053 ± 0.003	145.2 ± 26.9	0.37
#Ca2	0.312 ± 0.01	85.5 ± 9.5	3.65	0.263 ± 0.008	53.3 ± 6.1	4.94	#Ch2	0.036 ± 0.002	143.5 ± 26.6	0.25	0.029 ± 0.002	125.0 ± 21.3	0.23
#Ca3	1.062 ± 0.079	142.2 ± 32.0	7.47	0.871 ± 0.056	70.2 ± 16.7	12.4	#Ch3	0.037 ± 0.003	82.1 ± 22.0	0.45	0.027 ± 0.003	45.6 ± 18.7	0.59
#Ca4	0.351 ± 0.027	116.9 ± 28.4	3.00	0.172 ± 0.01	95.8 ± 18.9	1.80	#Ch4	0.087 ± 0.008	139.5 ± 33.5	0.62	0.062 ± 0.006	81.7 ± 24.2	0.76
#Ca5	0.184 ± 0.016	187.8 ± 46.0	0.98	0.128 ± 0.011	190.3 ± 43.1	0.67	#Ch5	0.053 ± 0.005	134.6 ± 36.9	0.39	0.042 ± 0.003	112.4 ± 28.2	0.37
#Ca6	0.489 ± 0.027	155.8 ± 25.0	3.14	0.396 ± 0.018	87.7 ± 13.9	4.52	#Ch6	0.047 ± 0.005	200.9 ± 59.4	0.23	0.037 ± 0.004	136.5 ± 48.5	0.27
#Ca7	0.559 ± 0.021	128.1 ± 15.3	4.36	0.459 ± 0.02	76.8 ± 12.0	5.98	#Ch7	0.038 ± 0.004	168.3 ± 48.3	0.23	0.026 ± 0.002	149.1 ± 28.1	0.17
#Ca8	0.183 ± 0.008	139.9 ± 18.5	1.31	0.148 ± 0.009	86.0 ± 17.5	1.73	#Ch8	0.058 ± 0.003	128.9 ± 18.3	0.45	0.043 ± 0.003	113.0 ± 23.3	0.38

#Ca9	0.198 ± 0.008	96.0 ± 13.8	2.08	0.221 ± 0.01	52.8 ± 8.9	4.18	#Ch9	0.056 ± 0.005	79.1 ± 23.6	0.70	0.037 ± 0.004	59.3 ± 22.4	0.62
#Ca10	0.318 ± 0.009	184.6 ± 14.0	1.72	0.248 ± 0.006	159.0 ± 10.6	1.56	#Ch10	0.063 ± 0.002	102.9 ± 10.3	0.61	0.044 ± 0.002	76.8 ± 10.3	0.57
#Ca11	0.391 ± 0.026	158.4 ± 30.6	2.47	0.263 ± 0.016	136.8 ± 25.3	1.92	#Ch11	0.060 ± 0.003	80.2 ± 14.5	0.74	0.037 ± 0.002	63.5 ± 13.3	0.59
#Ca12	0.245 ± 0.018	198.4 ± 40.6	1.23	0.174 ± 0.015	161.6 ± 40.0	1.07	#Ch12	0.045 ± 0.004	133.0 ± 32.4	0.34	0.026 ± 0.002	94.4 ± 22.7	0.28
#Ca13	0.892 ± 0.015	184.3 ± 8.7	4.84	0.707 ± 0.013	97.6 ± 6.0	7.25	#Ch13	0.083 ± 0.005	112.7 ± 21.3	0.74	0.062 ± 0.003	121.6 ± 20.7	0.51
#Ca14	1.323 ± 0.06	110.8 ± 16.3	11.94	1.364 ± 0.038	55.5 ± 6.0	24.6	#Ch14	0.050 ± 0.003	112.3 ± 23.0	0.45	0.040 ± 0.002	115.3 ± 22.4	0.35
#Ca15	0.757 ± 0.052	195.2 ± 36.5	3.88	0.618 ± 0.031	128.1 ± 19.9	4.82	#Ch15	0.109 ± 0.007	176.0 ± 33.7	0.62	0.082 ± 0.006	148.0 ± 32.9	0.56
#Ca16	0.244 ± 0.013	248.7 ± 34.2	0.98	0.190 ± 0.01	179.3 ± 26.0	1.06	#Ch16	0.151 ± 0.010	151.3 ± 30.1	1.00	0.105 ± 0.007	124.3 ± 25.7	0.84
#Ca17	0.205 ± 0.011	186.7 ± 28.9	1.10	0.143 ± 0.006	162.2 ± 21.2	0.88	#Ch17	0.036 ± 0.001	172.6 ± 17.8	0.21	0.043 ± 0.002	355.3 ± 40.5	0.12
#Ca18	0.150 ± 0.007	98.7 ± 15.8	1.52	0.146 ± 0.005	62.8 ± 8.0	2.33	#Ch18	0.073 ± 0.004	192.6 ± 31.9	0.38	0.061 ± 0.004	226.6 ± 35.3	0.27
#Ca19	0.286 ± 0.011	126.4 ± 15.0	2.26	0.188 ± 0.005	102.8 ± 9.7	1.83	#Ch19	0.091 ± 0.009	82.1 ± 29.4	1.11	0.061 ± 0.006	73.2 ± 24.6	0.83
#Ca20	0.105 ± 0.004	136 ± 15.7	0.77	0.080 ± 0.003	121.3 ± 13.0	0.66	#Ch20	0.014 ± 0.001	76.5 ± 15.4	0.18	0.009 ± 0.001	84.9 ± 39.1	0.11

#Ca21	0.256 ± 0.007	121.7 ± 10.6	2.11	0.285 ± 0.007	66.6 ± 6.5	4.27	#Ch21	0.102 ± 0.003	134.1 ± 12.8	0.76	0.065 ± 0.003	80.1 ± 12.0	0.81
#Ca22	0.201 ± 0.007	205.7 ± 19.5	0.97	0.165 ± 0.006	147.8 ± 15.7	1.12	#Ch22	0.102 ± 0.002	57.3 ± 27.0	1.78	0.066 ± 0.007	51.4 ± 22.2	1.29
#Ca23	0.127 ± 0.005	199.1 ± 22.7	0.64	0.093 ± 0.003	134.2 ± 13.1	0.69	#Ch23	0.059 ± 0.004	174.6 ± 35.1	0.34	0.035 ± 0.003	127.1 ± 28.4	0.28
#Ca24	0.268 ± 0.006	214.7 ± 12.6	1.25	0.189 ± 0.004	178.8 ± 13.4	1.06	#Ch24	0.019 ± 0.001	70.4 ± 17.4	0.27	0.013 ± 0.001	84.1 ± 18.4	0.16
#Ca25	0.858 ± 0.022	123.6 ± 9.9	6.94	0.921 ± 0.026	63.0 ± 6.7	14.6	#Ch25	0.057 ± 0.005	104.0 ± 28.6	0.54	0.041 ± 0.003	94.5 ± 21.2	0.44

[a] nmol/min/mg liver microsomes, [b] µM, [c] $V_{max}/K_m \times 1000$ µl/min/mg liver microsomes

Table S10 Prediction of unbound C_{max} of R- and S-methadone in the heart venous blood at steady-state after a repeated oral methadone enantiomer dose of 30 mg/day for 30 days in individuals by the individual PBK models using in vitro kinetic data obtained from individual microsomes and by the PBK model linked with Monte Carlo (MC) simulations based on variations in the metabolism using reported in vitro kinetic data of rCYPs

	Unbound C_{max} in the heart venous blood obtained from individual PBK models				Unbound C_{max} in the heart venous blood obtained from PBK model and MC simulation considering variations in the metabolism			
	R-methadone		S-methadone		R-methadone		S-methadone	
	Caucasian	Chinese	Caucasian	Chinese	Caucasian	Chinese	Caucasian	Chinese
Geometric mean (mg/day)	38.1	85.7	28.3	85.4	37.6	91.8	28.6	97.9
Geometric CV (%)	58	27	94	33	42	23	51	32
Fold difference	5.5 [a]	2.7[a]	10 [a]	3 [a]	4.6 [b]	2.9[b]	6.2 [b]	3.9 [b]

[a] fold differences between highest and lowest predicted unbound C_{max} in the heart venous blood in the population, [b] fold differences between 1st and 99th percentile for predicted unbound C_{max} in the heart venous blood in the population.

Table S11 Prediction of unbound C_{max} of R- and S-methadone in the heart venous blood at steady-state after a repeated oral methadone enantiomer dose of 30 mg/day for 30 days in individuals by the PBK model linked with Monte Carlo (MC) simulations based on variations in bodyweight, oral fraction absorbed, fraction unbound in plasma in addition to metabolism

	Unbound C_{max} in the heart venous blood obtained from PBK models and MC simulation considering variations in multiple parameters			
	R-methadone		S-methadone	
	Caucasian	Chinese	Caucasian	Chinese
Geometric mean (mg/day)	31.6	74.2	24.5	79.6
Geometric CV (%)	71	53	77	60
Fold difference	11.9 [a]	7.8[a]	13.7[a]	9.5 [a]

[a] fold differences between 1st and 99th percentile for predicted unbound C_{max} in the heart venous blood in the population.

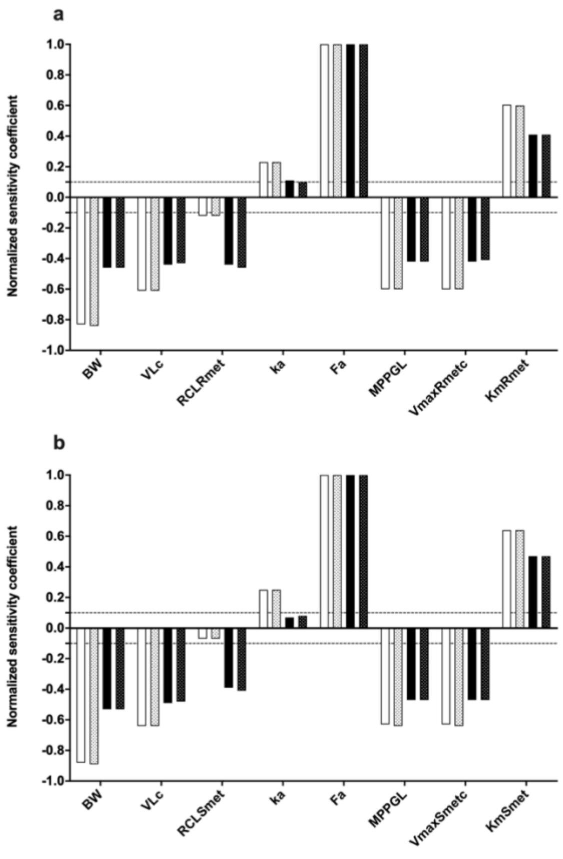

Fig. S1 SCs of PBK model parameters for the prediction of steady-state C_{max} of R-methadone **(a)** and S-methadone **(b)** in the heart venous blood upon the oral repeated methadone enantiomer doses of 10 mg enantiomers/day (white bars for Caucasians, black bars for Chinese) and 100 mg enantiomers/day (white bars with dots for Caucasians, black bars with dots for Chinese) for 30 days. BW, body weight; VLc, fraction of liver; RCLRmet and RCLSmet, renal clearance of R- and S-methadone; ka, absorption rate constant; Fa, oral fraction absorbed; MPPGL, microsomal protein per gram of liver; VmaxRmetc and VmaxSmetc, unscaled maximum rate of R- and S-methadone metabolism in liver; KmRmet and KmSmet, Michaelis-Menten constant for R- and S-methadone metabolism in liver

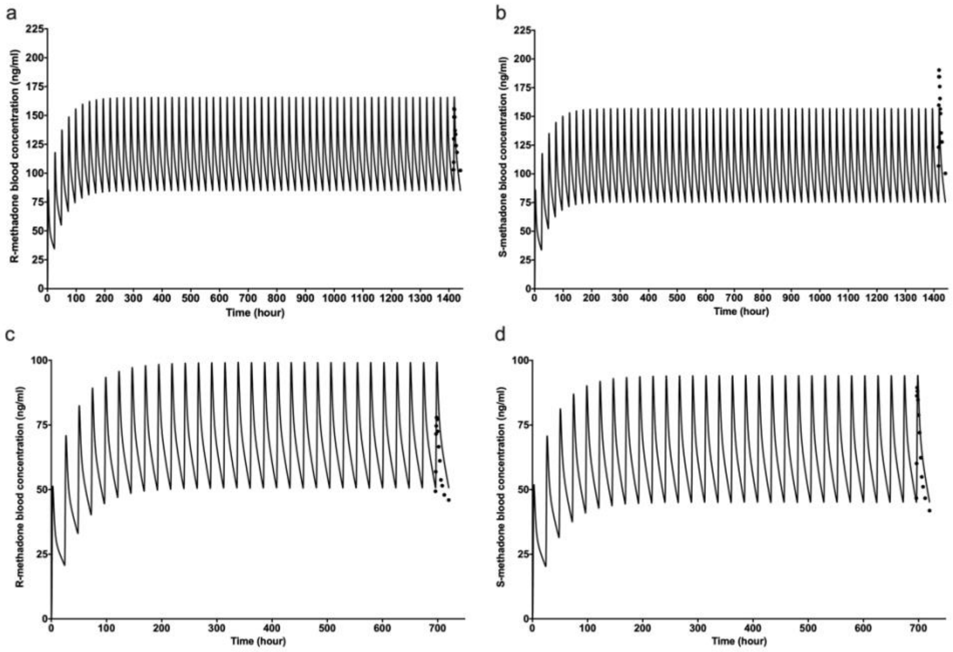

Fig. S2 Blood concentration-time curves of R- methadone **(a, c)** and S-methadone **(b, d)** in human predicted with the PBK model (lines) using HLM kinetic data and published in vivo data (dots) after a repeated oral rac-methadone dose of 70 mg/day for 60 days **(a, b)** (Foster et al., 2000) and 40 mg/day for 30 days **(c, d)** (Garimella et al., 2015)

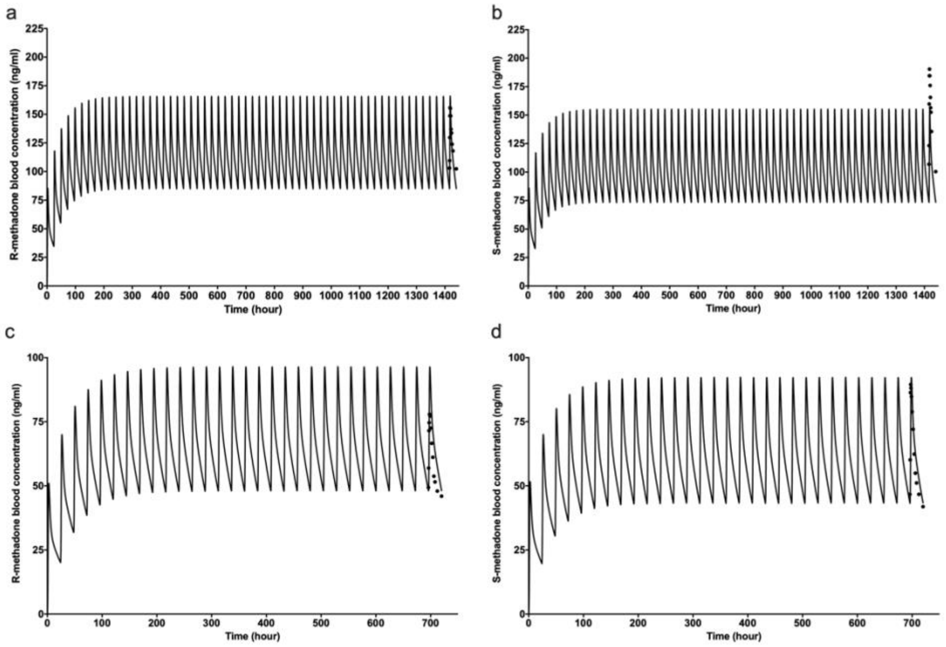

Fig. S3 Blood concentration-time curves of R- methadone **(a, c)** and S-methadone **(b, d)** in human predicted with the PBK model (lines) using rCYPs kinetic data and published in vivo data (dots) after a repeated oral rac-methadone dose of 70 mg/day for 60 days **(a, b)** (Foster et al., 2000) and 40 mg/day for 30 days **(c, d)** (Garimella et al., 2015)

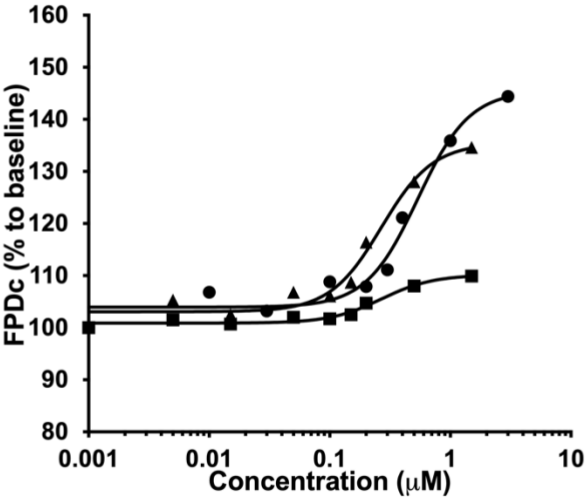

Fig. S4 Concentration-response curves for the effect of rac-methadone (circles), R-methadone (squares) and S-methadone (triangles) on corrected field potential duration (FPDc) in human induced pluripotent stem cell derived cardiomyocytes detected by the multielectrode array. The concentration-response curve of rac-methadone (Shi et al., 2020a) was corrected to the curve of R- and S-methadone based on the potency difference between R- and S-methadone in blocking potassium channels as reported in Eap et al., (2007)

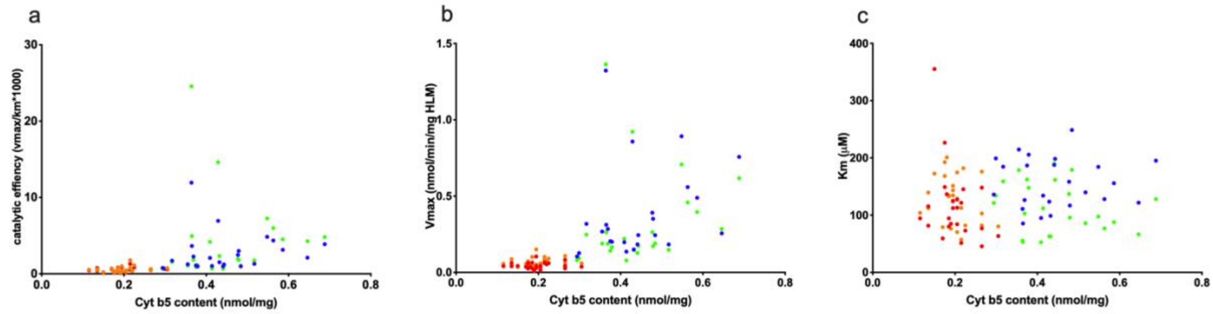

Fig. S5 Correlation between Cytochrome b5 (Cyt b5) content and catalytic efficiency **(a)**, V_{max} **(b)** and K_m **(c)**. Dots in blue, green, orange and red represent the data for R-methadone in Caucasians, for S-methadone in Caucasians, R-methadone in Chinese and S-methadone in Chinese, respectively. The correlation coefficients (r) for the correlations are as follows: r=0.79**** for R-methadone; r=0.79**** for S-methadone **(a)**, r=0.82**** for R-methadone; r=0.8**** for S-methadone **(b)** and r=0.19 for R-methadone; r=-0.06 for S-methadone **(c)**. The data distribution was checked using Kolmogorov-Smirnov normality test. Since most data sets were log-normally distributed, nonparametric Spearman's correlation was used. A p value < 0.05 was regarded as statistically significant. The r values are marked with * with $p < 0.05$: *, $p < 0.01$: **, $p < 0.001$: *** and $p < 0.0001$: **** Statistical analysis was performed by Graph Pad Prism 8.0 (GraphPad Software Inc.)

Supplementary materials 2

Model code for Caucasian population without Monte Carlo simulation. The enantiomeric interaction equations are shown in *italic*.

```
;Model code
;==========================================================================================
; Physiological parameters
;==========================================================================================

;Tissue volumes (L or Kg)

BW = 70                              ; body weight human in kg (Brown et al., 1997)

; all fractions taken from Brown et al. (1997)
 VLc = 0.0257                        ; fraction of liver tissue
 VFc = 0.2142                        ; fraction of fat tissue
 VLuc =   0.0076                     ; fraction of lung tissue
 VAc =  0.0198                       ; fraction of arterial blood: 0.074*1/4
 VVc = 0.0593                        ; fraction of venous blood: 0.074*3/4
 VKc = 0.004                         ; fraction of kidney tissue
 VHc = 0.0047                        ; fraction of heart tissue
 VRc = 0.09-VLc - VLuc - VKc - VHc   ; fraction of richly perfused tissue
 VSc = 0.746-VFc                     ; Fraction of blood flow to slowly perfused tissue
                                     ; total of fractions = 0.9151

VL = VLc * BW                        ; volume of liver
VF = VFc * BW                        ; volume of fat
VLu = VLuc * BW                      ; volume of lungs
VK = VKc * BW                        ; volume of kidneys
VH = VHc * BW                        ; volume of heart
VR = VRc * BW                        ; volume of richly perfused tissue
VS = VSc * BW                        ; volume of slowly perfused tissue
VA = VAc * BW                        ; volume of arterial blood
VV = VVc * BW                        ; volume of venous blood
;-------------------------------------------------------------------------------------------
----------------------------------
;Blood flow rates (L/h)
  QC = 15 * BW^0.74                  ; Info: QC = 15 * BW^0.74  (Brown, 1997)
  QLc = 0.227                        ; Fraction of blood flow to liver
  QFc = 0.052                        ; Fraction of blood flow to fat
  QKc = 0.175                        ; fraction of blood flow to kidneys
  QHc = 0.04                         ; fraction of blood flow to heart
  QSc = 0.24-QFc                     ; Fraction of blood flow to slowly perfused tissue
  QRc = 0.76-QLc-QKc-QHc             ; fraction of blood flow to rapidly perfused tissue
                                     ; total of fractions = 1

; all fractions taken from Brown 1997
QL = QLc*QC                          ; blood flow rate to liver in L/hr
QF = QFc*QC                          ; blood flow rate to fat
```

```
QK = QKc * QC                 ; blood flow rate to kidneys
QH = QHc*QC                   ; blood flow rate to heart
QR = QRc*QC                   ; blood flow rate to richly perfused tissue
QS = QSc*QC                   ; blood flow rate to slowly perfused tissue

;==============================================================================
; Partition Coefficients
;==============================================================================
; R-Methadone
PLRmet = 12.53                ; liver/blood partition coefficient R-Methadone
PFRmet = 3.33                 ; fat/blood partition coefficient R-Methadone
PRRmet = 12.53                ; richly perfused tissues/blood partition coefficient R-Methadone
PSRmet = 7.71                 ; slowly perfused tissues/blood partition coefficient R-Methadone
PLuRmet = 1.77                ; lung/blood partition coefficient R-Methadone
PKRmet = 7.6                  ; kidney/blood partition coefficient R-Methadone
PHRmet = 4.93                 ; heart/blood partition coefficient R-Methadone

; S-Methadone
PLSmet = 11.99                ; liver/blood partition coefficient S-Methadone
PFSmet = 2.54                 ; fat/blood partition coefficient S-Methadone
PRSmet = 11.99                ; richly perfused tissues/blood partition coefficient S-Methadone
PSSmet = 7.39                 ; slowly perfused tissues/blood partition coefficient S-Methadone
PLuSmet = 1.71                ; lung/blood partition coefficient S-Methadone
PKSmet = 7.29                 ; kidney/blood partition coefficient S-Methadone
PHSmet = 4.73                 ; heart/blood partition coefficient S-Methadone

;=====================================================================================
; Biochemical parameters
;=====================================================================================

;Linear uptake rate (/h)
ka = 0.59                     ; obtained from Foster et al. (2000); Wolff et al. (2000)

;Fraction absorbed
Fa = 0.88                     ; obtained from Ke et al. (2013)

;Renal clearance (L/h)
RCLRmet =1.8                  ; average values obtained from Boulton et al. (2001); Kharasch et al. (2009)
Foster et al. (2000)

RCLSmet =1.1                  ; average values obtained from Boulton et al. (2001); Kharasch et al. (2009)
Foster et al. (2000)

;------------------------------------------------------------------------------------------------------------------------------------------
;Metabolism liver

;Scaling factors
ISEFCYP2B6R=0.13              ; corrected based on Totah et al. (2007) (2008)
ISEFCYP3A4R=0.04              ; corrected based on Totah et al. (2007) (2008)
```

ISEFCYP2C19R= 0.1 ; corrected based on Totah et al. (2007) (2008)

ISEFCYP2B6S=0.13 ; corrected based on Totah et al. (2007) (2008)
ISEFCYP3A4S=0.03 ; corrected based on Totah et al. (2007) (2008)
ISEFCYP2C19S= 0.39 ; corrected based on Totah et al. (2007) (2008)

aCYP2B6 = 17 ; EM CYP abundance level pmol/mg CYPisoform from Barter et al. (2013)
;aCYP2B6 =6 ; PM CYP abundance level pmol/mg CYPisoform from Barter et al. (2013)
aCYP3A4 = 93 ; CYP abundance level pmol/mg CYPisoform from Achour et al. (2014)
n=713
aCYP2C19 = 11 ; CYP abundance level pmol/mg CYPisoform from Achour et al. (2014),
n=76;

MPL=32 ; liver microsomal protein yield (mg/gram liver) (Barter et al., 2007)
L=VLc*1000 ; liver = 25.7 (gram/kg BW)

;in vitro recombinant incubation of R-methadone (pmol/min/nmol CYP)
VmaxRmetCYP2B6m = 36 ; Totah et al. (2007)
VmaxRmetCYP3A4m = 43 ; Totah et al. (2007)
VmaxRmetCYP2C19m = 22 ; Totah et al. (2007)

;metabolites of R-methadone, unscaled maximum rate of metabolism (pmol/mg protein/min)
VmaxRmetCYP2B6c = VmaxRmetCYP2B6m*ISEFCYP2B6R*aCYP2B6
VmaxRmetCYP3A4c = VmaxRmetCYP3A4m*ISEFCYP3A4R*aCYP3A4
VmaxRmetCYP2C19c = VmaxRmetCYP2C19m*ISEFCYP2C19R*aCYP2C19

;metabolites of R-Methadone, scaled maximum rate of metabolism (µmol/h)
VMaxRmetCYP2B6 = VmaxRmetCYP2B6c / 1000000 * 60 * MPL * L * BW
VMaxRmetCYP3A4 = VmaxRmetCYP3A4c / 1000000 * 60 * MPL * L * BW
VMaxRmetCYP2C19= VmaxRmetCYP2C19c / 1000000 * 60 * MPL * L * BW

;metabolites of R-methadone, affinity constants (µmol/L)
KmRmetCYP2B6 = 60
KmRmetCYP3A4 = 137
KmRmetCYP2C19 = 97

;in vitro recombinant incubation of S-methadone (pmol/min/nmol CYP)
VmaxSmetCYP2B6m = 15 ; Totah et al. (2007)
VmaxSmetCYP3A4m = 46 ; Totah et al. (2007)
VmaxSmetCYP2C19m = 8 ; Totah et al. (2007)

;metabolites of S-methadone, unscaled maximum rate of metabolism (pmol/mg protein/min)
VmaxSmetCYP2B6c = VmaxSmetCYP2B6m*ISEFCYP2B6S*aCYP2B6
VmaxSmetCYP3A4c = VmaxSmetCYP3A4m*ISEFCYP3A4S*aCYP3A4
VmaxSmetCYP2C19c = VmaxSmetCYP2C19m*ISEFCYP2C19S*aCYP2C19

; metabolites of S-methadone, scaled maximum rate of metabolism (µmol/h)
VMaxSmetCYP2B6 = VmaxSmetCYP2B6c / 1000000 * 60 * MPL * L * BW
VMaxSmetCYP3A4 = VmaxSmetCYP3A4c / 1000000 * 60 * MPL * L * BW

```
VMaxSmetCYP2C19 = VmaxSmetCYP2C19c / 1000000 * 60 * MPL * L * BW

 ;metabolites of S-methadone, affinity constants (umol/L)
KmSmetCYP2B6 = 16
KmSmetCYP3A4 = 149
KmSmetCYP2C19 = 125

;====================================================================================
;Run settings
;====================================================================================

;molecular weight (g/mol)
	MWRmet= 309.4			; molecular weight
	MWSmet= 309.4			; molecular weight

; R-methadone Given dose (mg/kg bw) and oral dose in µmol/kg bw
	TDOSERmet = 30			; whole body total dose in mg
	GDOSERmet = TDOSERmet / BW	; given dose in mg per kg bw
	ODOSERmet = GDOSERmet * 1e-3 / MWRmet *1e6	; determine odose (µmol/kg bw)
	DOSERmet = ODOSERmet * BW			; determine dose in µmol


; S-methadone Given dose (mg/kg bw) and oral dose in µmol/kg bw
	TDOSESmet = 30			; whole body total dose in mg
	GDOSESmet = TDOSESmet / BW	; given dose in mg per kg bw
	ODOSESmet = GDOSESmet * 1e-3 / MWSmet *1e6	; determine odose (µmol/kg bw)
	DOSESmet = ODOSESmet * BW			; determine dose in µmol


	dose_int = 24					; dosing interval in hours
;Time
	Starttime = 0					; in hrs
	Stoptime = 30*24				; in hrs (days * hours in a day)
	DTMIN = 1e-6
	DTMAX = 1
	DTOUT = 0
	TOLERANCE = 0.00001

;====================================================================================
;Kinetics
;====================================================================================
;slowly perfused tissue compartment

;ASRmet = Amount R-methadone in slowly perfused tissue (µmol)
    ASRmet' = QS * (CARmet - CVSRmet)
    Init ASRmet = 0
    CSRmet = ASRmet / VS
    CVSRmet = CSRmet / PSRmet
```

```
;ASSmet = Amount S-methadone in slowly perfused tissue (µmol)
      ASSmet' = QS * (CASmet - CVSSmet)
      Init ASSmet = 0
      CSSmet = ASSmet / VS
      CVSSmet = CSSmet / PSSmet

;----------------------------------------------------------------------------------------------------------------------------------------------------
---------------------------------
; rapid perfused tissue compartment

;ARRmet = Amount R-methadone in richly perfused tissue (µmol)
      ARRmet' = QR * (CARmet - CVRRmet)
      Init ARRmet = 0
      CRRmet = ARRmet / VR
      CVRRmet = CRRmet / PRRmet

;ARSmet = Amount S-methadone in richly perfused tissue (µmol)
      ARSmet' = QR * (CASmet - CVRSmet)
      Init ARSmet = 0
      CRSmet = ARSmet / VR
      CVRSmet = CRSmet / PRSmet
;----------------------------------------------------------------------------------------------------------------------------------------------------
---------------------------------
;fat compartment

;AFRmet = Amount R-methadone in fat tissue (µmol)
      AFRmet' = QF * (CARmet - CVFRmet)
      Init AFRmet = 0
      CFRmet = AFRmet / VF
      CVFRmet = CFRmet / PFRmet

;AFSmet = Amount S-methadone in fat tissue (µmol)
      AFSmet' = QF * (CASmet - CVFSmet)
      Init AFSmet = 0
      CFSmet = AFSmet / VF
      CVFSmet = CFSmet / PFSmet
;----------------------------------------------------------------------------------------------------------------------------------------------------
; uptake methadone from GI tract

;AGIRmet = Amount R-methadone remaining in GI tract (µmol)

       Init AGIRmet = 0
       AGIRmet' = pulse(DOSERmet* Fa, 0, dose_int) + AGIRmet * -Ka

;AGISmet = Amount S-methadone remaining in GI tract (µmol)

       Init AGISmet = 0
       AGISmet' = pulse(DOSESmet* Fa, 0, dose_int) + AGISmet * -Ka
```

```
;---------------------------------------------------------------------------------------------------------------------------------------------------------------------------------------
;liver compartment

;ALRmet = Amount R-methadone in liver tissue (µmol)
       ALRmet' =  QL * (CARmet - CVLRmet )+ (AGIRmet * Ka) - AMLRmetCYP2B6' -AMLRmetCYP3A4' - AMLRmetCYP2C19'
      Init ALRmet = 0
      CLRmet = ALRmet / VL
      CVLRmet = CLRmet / PLRmet

;metabolism described by Michaelis-Menten Kinetics
     ;AMLRmetCYP2B6=Amount R-methadone metabolized in liver to R-EDDP by CYP2B6
     ;AMLRmetCYP2B6' = (VmaxRmetCYP2B6*CVLRmet)  / (KmRmetCYP2B6 + CVLRmet)
     ;init AMLRmetCYP2B6 = 0

     ;AMLRmetCYP3A4=Amount R-methadone metabolized in liver to R-EDDP by CYP3A4
     ;AMLRmetCYP3A4' = (VmaxRmetCYP3A4*CVLRmet)  / (KmRmetCYP3A4 + CVLRmet)
     ;init AMLRMetCYP3A4 = 0

     ;AMLRmetCYP2C19=Amount R-methadone metabolized in liver to R-EDDP by CYP2C19
     ;AMLRmetCYP2C19' = (VmaxRmetCYP2C19*CVLRmet)  / (KmRmetCYP2C19 + CVLRmet)
     ;init AMLRMetCYP2C19 = 0

;metabolism described by enantiomeric interactions equations

     AMLRmetCYP2B6'=VmaxRmetCYP2B6*((CVLRmet*CVLRmet/(ahCYP2B6*KmRmetCYP2B6*KmRmetCYP2B6))+(CVLRmet/KmRmetCYP2B6)+(CVLRmet*CVLSmet/(bhCYP2B6*KmRmetCYP2B6*KmSmetCYP2B6)))/(1+(CVLRmet*CVLRmet/(ahCYP2B6*KmRmetCYP2B6*KmRmetCYP2B6))+(2*CVLRmet/KmRmetCYP2B6)+(CVLSmet*CVLSmet/(ahCYP2B6*KmSmetCYP2B6*KmSmetCYP2B6))+(2*CVLSmet/KmSmetCYP2B6)+(2*CVLRmet*CVLSmet/(bhCYP2B6*KmRmetCYP2B6*KmSmetCYP2B6)))

     ahCYP2B6=5                    ; homotropic interaction factor (Totah et al., 2007)
     bhCYP2B6=7                    ; heterotropic interaction factor (Totah et al., 2007)

     init AMLRmetCYP2B6 = 0

     AMLRmetCYP3A4' =
     (VmaxRmetCYP3A4*((CVLRmet*CVLRmet/(ahCYP3A4*KmRmetCYP3A4*KmRmetCYP3A4))+(CVLRmet/KmRmetCYP3A4)))/(1+(CVLRmet*CVLRmet/(ahCYP3A4*KmRmetCYP3A4*KmRmetCYP3A4))+(2*CVLRmet/KmRmetCYP3A4)+(CVLSmet*CVLSmet/(ahCYP3A4*KmSmetCYP3A4*KmSmetCYP3A4))+(2*CVLSmet/KmSmetCYP3A4)+(2*CVLRmet*CVLSmet/(bhCYP3A4*KmRmetCYP3A4*KmSmetCYP3A4)))

     ahCYP3A4=4                    ; homotropic interaction factor (Totah et al., 2007)
     bhCYP3A4=2                    ; heterotropic interaction factor (Totah et al., 2007)

     init AMLRmetCYP3A4 = 0
```

AMLRmetCYP2C19' = (VmaxRmetCYP2C19(CVLRmet/KmRmetCYP2C19))/(1+(CVLRmet*CVLRmet/(ahCYP2C19*KmRmetCYP2C19*KmRmetCYP2C19))+(2*CVLRmet/KmRmetCYP2C19)+(CVLSmet*CVLSmet/(ahCYP2C19*KmSmetCYP2C19*KmSmetCYP2C19))+(2*CVLSmet/KmSmetCYP2C19)+(2*CVLRmet*CVLSmet/(bhCYP2C19*KmRmetCYP2C19*KmSmetCYP2C19)))*

ahCYP2C19=42 ; homotropic interaction factor (Totah et al., 2007)
bhCYP2C19=3 ; heterotropic interaction factor (Totah et al., 2007)

init AMLRmetCYP2C19 = 0

;---
;S-methadone
;ALSmet = Amount S-methadone in liver tissue (µmol)
ALSmet' = QL * (CASmet - CVLSmet)+ (AGISmet * Ka) - AMLSmetCYP2B6' -AMLSmetCYP3A4' - AMLSmetCYP2C19'
Init ALSmet = 0
CLSmet = ALSmet / VL
CVLSmet = CLSmet / PLSmet

;metabolism described by Michaelis-Menten Kinetics
;AMLSmetCYP2B6=Amount Smet metabolized in liver to S-EDDP by CYP2B6
;AMLSmetCYP2B6' = (VmaxSmetCYP2B6*CVLSmet) / (KmSmetCYP2B6 + CVLSmet)
;init AMLSmetCYP2B6 = 0

;AMLSmetCYP3A4=Amount Smet metabolized in liver to S-EDDP by CYP3A4
;AMLSmetCYP3A4' = (VmaxSmetCYP3A4*CVLSmet) / (KmSmetCYP3A4 + CVLSmet)
;init AMLSmetCYP3A4 = 0

;AMLSmetCYP2C19=Amount Smet metabolized in liver to S-EDDP by CYP2C19
;AMLSmetCYP2C19' = (VmaxSmetCYP2C19*CVLSmet) / (KmSmetCYP2C19 + CVLSmet)
;init AMLSMetCYP2C19 = 0

;metabolism described by enantiomeric interactions equations

AMLSmetCYP2B6' = VmaxSmetCYP2B6((CVLSmet*CVLSmet/(ahCYP2B6*KmSmetCYP2B6*KmSmetCYP2B6))+(CVLSmet/KmSmetCYP2B6)+(CVLRmet*CVLSmet/(bhCYP2B6*KmRmetCYP2B6*KmSmetCYP2B6)))/(1+(CVLRmet*CVLRmet/(ahCYP2B6*KmRmetCYP2B6*KmRmetCYP2B6))+(2*CVLRmet/KmRmetCYP2B6)+(CVLSmet*CVLSmet/(ahCYP2B6*KmSmetCYP2B6*KmSmetCYP2B6))+(2*CVLSmet/KmSmetCYP2B6)+(2*CVLRmet*CVLSmet/(bhCYP2B6*KmRmetCYP2B6*KmSmetCYP2B6)))*

ahCYP2B6=5 ; homotropic interaction factor (Totah et al., 2007)
bhCYP2B6=7 ; heterotropic interaction factor (Totah et al., 2007)

init AMLSmetCYP2B6 = 0

AMLSmetCYP3A4' = (VmaxSmetCYP3A4((CVLSmet*CVLSmet/(ahCYP3A4*KmSmetCYP3A4*KmSmetCYP3A4))+(CVLSmet/K*

```
mSmetCYP3A4)))/(1+(CVLRmet*CVLRmet/(ahCYP3A4*KmRmetCYP3A4*KmRmetCYP3A4))+(2*CVLRme
t/KmRmetCYP3A4)+(CVLSmet*CVLSmet/(ahCYP3A4*KmSmetCYP3A4*KmSmetCYP3A4))+(2*CVLSmet/
KmSmetCYP3A4)+(2*CVLRmet*CVLSmet/(bhCYP3A4*KmRmetCYP3A4*KmSmetCYP3A4)))

ahCYP3A4=4                    ; homotropic interaction factor (Totah et al., 2007)
bhCYP3a4=2                    ; heterotropic interaction factor (Totah et al., 2007)

init AMLSmetCYP3A4 = 0

AMLSmetCYP2C19' =
(VmaxSmetCYP2C19*(CVLSmet/KmSmetCYP2C19))/(1+(CVLRmet*CVLRmet/(ahCYP2C19*KmRmetCYP
2C19*KmRmetCYP2C19))+(2*CVLRmet/KmRmetCYP2C19)+(CVLSmet*CVLSmet/(ahCYP2C19*KmSmetC
YP2C19*KmSmetCYP2C19))+(2*CVLSmet/KmSmetCYP2C19)+(2*CVLRmet*CVLSmet/(bhCYP2C19*KmR
metCYP2C19*KmSmetCYP2C19)))

ahCYP2C19=42                  ; homotropic interaction factor (Totah et al., 2007)
bhCYP2C19=3                   ; heterotropic interaction factor (Totah et al., 2007)

init AMLSmetCYP2C19 = 0

;------------------------------------------------------------------------------------------------------------------------------------------------
;kidney compartment

;AKRmet = Amount R-methadone in kidney tissue (µmol)
    AKRmet' =  QK * (CARmet - CVKRmet)- ACLRmet'
    Init AKRmet = 0
    CKRmet = AKRmet / VK
    CVKRmet = CKRmet / PKRmet

;ACLRmet=Amount R-methadone cleared renally
    ACLRmet'=RCLRmet*CVKRmet
    init ACLRmet = 0


;AKSmet = Amount S-methadone in kidney tissue (µmol)
    AKSmet' =  QK * (CASmet - CVKSmet)- ACLSmet'
    Init AKSmet = 0
    CKSmet = AKSmet / VK
    CVKSmet = CKSmet / PKSmet

;ACLSmet=Amount S-methadone cleared renally
    ACLSmet'=RCLSmet*CVKSmet
    init ACLSmet = 0

;------------------------------------------------------------------------------------------------------------------------------------------------
----------------------------------
;Heart compartment

;AHRmet = Amount R-methadone in heart tissue (µmol)
```

```
	AHRmet' = QH * (CARmet - CVHRmet)
	Init AHRmet = 0
	CHRmet = AHRmet / VH
	CVHRmet = CHRmet / PHRmet

;AHSmet = Amount S-methadone in heart tissue (µmol)
	AHSmet' = QH * (CASmet - CVHSmet)
	Init AHSmet = 0
	CHSmet = AHSmet / VH
	CVHSmet = CHSmet / PHSmet

;-----------------------------------------------------------------------------------------------------------------------------------------------
;lung compartment

;ALuRmet = Amount R-methadone in lung tissue (µmol)
	ALuRmet' = QC * (CVRmet - CALuRmet)
	Init ALuRmet = 0
	CLuRmet = ALuRmet / VLu
	CALuRmet = CLuRmet / PLuRmet


;ALuSmet = Amount S-methadone in lung tissue (µmol)
	ALuSmet' = QC * (CVSmet - CALuSmet)
	Init ALuSmet = 0
	CLuSmet = ALuSmet / VLu
	CALuSmet = CLuSmet / PLuSmet

;-----------------------------------------------------------------------------------------------------------------------------------------------
---------------------------------
; arterial blood compartment

;CARmet = Concentration arterial blood R-methadone
	AARmet' = QC * (CALuRmet- CARmet);
	Init AARmet = 0
	CARmet=  AARmet / VA

;CASmet = Concentration arterial blood S-methadone
	AASmet' = QC * (CALuSmet- CASmet);
	Init AASmet = 0
	CASmet=  AASmet / VA

;-----------------------------------------------------------------------------------------------------------------------------------------------
---------------------------------
; venous blood compartment

;AVRmet = amount venous blood R-methadone (µmol)
	AVRmet' = (QF * CVFRmet + QR * CVRRmet + QS * CVSRmet + QL * CVLRmet + QK * CVKRmet + QH 
*CVHRmet- QC * CVRmet)
	Init AVRmet = 0
```

```
      CVRmet = (AVRmet / VV)


;AVSmet = amount venous blood S-methadone (µmol)
      AVSmet' = (QF * CVFSmet + QR * CVRSmet + QS * CVSSmet + QL * CVLSmet + QK * CVKSmet + QH
*CVHSmet- QC * CVSmet)
      Init AVSmet = 0
      CVSmet = (AVSmet / VV)

;=====================================================================================
;Mass balance calculations
;=====================================================================================
{Mass Balance}
TotalRMet' = pulse(DOSERmet *Fa, 0, dose_int)
init TotalRmet = 1E-50

CalculatedRmet = AFRMet + ASRmet+ ARRmet + ALRmet+ AVRmet+ AARmet + AGIRmet + AMLRmetCYP2B6 +
AMLRmetCYP3A4 + AMLRmetCYP2C19+ALuRmet + AKRmet + AHRmet + ACLRmet


ERRORRmet = ((TotalRmet - CalculatedRmet) / (TotalRmet + 1E-30)) * 100
MASSBALRmet = TotalRmet - CalculatedRmet + 1

;-------------------------------------------------------------------------------------------------------------------------------------------

TotalSMet' = pulse(DOSESmet *Fa, 0, dose_int)
init TotalSmet = 1E-50

CalculatedSmet = AFSMet + ASSmet+ ARSmet + ALSmet+ AVSmet+ AASmet + AGISmet + AMLSmetCYP2B6 +
AMLSmetCYP3A4 + AMLSmetCYP2C19 + ALuSmet + AKSmet + AHSmet + ACLSmet


ERRORSmet = ((TotalSmet - CalculatedSmet) / (TotalSmet + 1E-30)) * 100
MASSBALSmet = TotalSmet - CalculatedSmet + 1

;=====================================================================================
;Calculation with model
;=====================================================================================

CVRmetB = CVRmet * MWRmet                ; concentration of R-methadone in venous blood (µg/L)
AUCRmet' = CVRmetB                       ; calculate AUC for R-methadone
init AUCRmet = 0

CVSmetB = CVSmet * MWSmet                ; concentration of S-methadone in venous blood (µg/L)
AUCSmet' = CVSmetB                       ; calculate AUC for S-methadone
init AUCSmet = 0
;-----------------------------------------------------------------------------------------------
CVheartRmet= CVHRmet*MWRmet              ; concentration of R-methadone in the heart venous blood (µg/L)
CVheartSmet= CVHSmet*MWSmet              ; concentration of S-methadone in the heart venous blood (µg/L)
```

;--

fupRmet=0.16 ; unbound fraction in plasma of R-methadone (Ke et al., 2014)

fupSmet=0.12 ; unbound fraction in plasma of S-methadone (Ke et al., 2014)

BRrRmet=0.7 ; blood to plasma ratio of rac-methadone (Hsu et al., 2013), assumed to be same for the two enantiomers (Badhan et al., 2019)

BRrSmet=0.7 ; blood to plasma ratio of rac-methadone (Hsu et al., 2013), assumed to be same for the two enantiomers (Badhan et al., 2019)

fCVheartRmet= CVheartRmet* (fupRmet/BPrRmet) ; unbound concentration of R-methadone in the heart venous blood (µg/L) according to (Shi et al., 2020a)

fCVheartSmet= CVheartSmet*(fupSmet/BPrSmet) ; unbound concentration of R-methadone in the heart venous blood (µg/L) according to (Shi et al., 2020a)

Supplementary materials 3

Model code of Monte Carlo simulation for Caucasian population

;Variations in metabolism

;CYP2B6 EM

;aCYP2B6c = init(exp(normal(2.38, 0.955)))

;aCYP2B6 = IF aCYP2B6c >0.61 AND aCYP2B6c < 189.01 THEN aCYP2B6c ELSE 100001 ;Values higher or lower than 3 times the SD are removed

;CYP2B6 PM

aCYP2B6c = init(exp(normal(0.99, 1.269)))

aCYP2B6 = IF aCYP2B6c >0.06 AND aCYP2B6c < 120.66 THEN aCYP2B6c ELSE 100001; Values higher or lower than 3 times the SD are removed

;CYP3A4 general population n=713

aCYP3A4c = init(exp(normal(4.28, 0.71)))

aCYP3A4 = IF aCYP3A4c >8.58 AND aCYP3A4c < 608.6 THEN aCYP3A4c ELSE 100001; Values higher or lower than 3 times the SD are removed

;CYP2C19 general population n=76

aCYP2C19c = init(exp(normal(2.14, 0.717)))

aCYP2C19 = IF aCYP2C19c >0.99 AND aCYP2C19c < 73.12 THEN aCYP2C19c ELSE 100001; Values higher or lower than 3 times the SD are removed

;Variation in body weight

BWc= init(exp(normal(4.21, 0.29)))

BW = IF BWc >27.79 AND BWc < 161.8 THEN BWc ELSE 100001

;Variation in the oral fraction absorbed

Fac= init(exp(normal(-0.17, 0.29)))

Fa = IF Fac >0.35 AND Fac < 1.000001 THEN Fac ELSE 100001

;Variation in fraction unbound in plasma

```
fupRmetc= init(exp(normal(-1.88, 0.29)))
fupRmet = IF fupRmetc >0.06 AND fupRmetc < 0.4 THEN fupRmetc ELSE 100001

fupSmetc= init(exp(normal(-2.16, 0.29)))
fupSmet = IF fupSmetc >0.05 AND fupSmetc < 0.3 THEN fupSmetc ELSE 100001
```
